# Identification of HNRNPK as Regulator of Hepatitis C Virus Particle Production

**DOI:** 10.1371/journal.ppat.1004573

**Published:** 2015-01-08

**Authors:** Marion Poenisch, Philippe Metz, Hagen Blankenburg, Alessia Ruggieri, Ji-Young Lee, Daniel Rupp, Ilka Rebhan, Kathrin Diederich, Lars Kaderali, Francisco S. Domingues, Mario Albrecht, Volker Lohmann, Holger Erfle, Ralf Bartenschlager

**Affiliations:** 1 Department of Infectious Diseases, Molecular Virology, Heidelberg University, Heidelberg, Germany; 2 Department of Computational Biology and Applied Algorithmics, Max Planck Institute for Informatics, Saarbrücken, Germany; 3 Center for Biomedicine, European Academy Bozen/Bolzano (EURAC), Bolzano, Italy, Affiliated Institute of the University of Lübeck, Lübeck, Germany; 4 ViroQuant Research Group Modeling, University of Heidelberg, Heidelberg, Germany; 5 Institute for Medical Informatics and Biometry, Medical Faculty Carl Gustav Carus, Technische Universität Dresden, Dresden, Germany; 6 ViroQuant-CellNetworks RNAi Screening Facility, University of Heidelberg, Heidelberg, Germany; The University of Chicago, United States of America

## Abstract

Hepatitis C virus (HCV) is a major cause of chronic liver disease affecting around 130 million people worldwide. While great progress has been made to define the principle steps of the viral life cycle, detailed knowledge how HCV interacts with its host cells is still limited. To overcome this limitation we conducted a comprehensive whole-virus RNA interference-based screen and identified 40 host dependency and 16 host restriction factors involved in HCV entry/replication or assembly/release. Of these factors, heterogeneous nuclear ribonucleoprotein K (HNRNPK) was found to suppress HCV particle production without affecting viral RNA replication. This suppression of virus production was specific to HCV, independent from assembly competence and genotype, and not found with the related Dengue virus. By using a knock-down rescue approach we identified the domains within HNRNPK required for suppression of HCV particle production. Importantly, HNRNPK was found to interact specifically with HCV RNA and this interaction was impaired by mutations that also reduced the ability to suppress HCV particle production. Finally, we found that in HCV-infected cells, subcellular distribution of HNRNPK was altered; the protein was recruited to sites in close proximity of lipid droplets and colocalized with core protein as well as HCV plus-strand RNA, which was not the case with HNRNPK variants unable to suppress HCV virion formation. These results suggest that HNRNPK might determine efficiency of HCV particle production by limiting the availability of viral RNA for incorporation into virions. This study adds a new function to HNRNPK that acts as central hub in the replication cycle of multiple other viruses.

## Author Summary

As obligate intracellular parasites with limited gene coding capacity viruses exploit host cell machineries for the sake of efficient replication and spread. Thus, identification of these cellular machineries and factors is necessary to understand how a given virus achieves efficient replication and eventually causes host cell damage. Hepatitis C virus (HCV) is an RNA virus replicating in the cytoplasm of hepatocytes. While viral proteins have been studied in great detail, our knowledge about how host cell factors are used by HCV for efficient replication and spread is still scarce. In the present study we conducted a comprehensive RNA-interference-based screen and identified 40 genes that promote the HCV lifecycle and 16 genes that suppress it. Follow-up studies revealed that one of these genes, the heterogeneous nuclear ribonucleoprotein K (HNRNPK), selectively suppresses production of infectious HCV particles. We mapped the domains of HNRNPK required for this suppression and demonstrate that this protein selectively binds to the HCV RNA genome. Based on the correlation between suppression of virus production, HCV RNA binding and recruitment to lipid droplets, we propose that HNRNPK might limit the amount of viral RNA genomes available for incorporation into virus particles. This study provides novel insights into the complexity of reactions that are involved in the formation of HCV virions.

## Introduction

Hepatitis C virus (HCV) is a major cause of liver disease affecting ∼130 million people worldwide [1]. Chronic HCV infection can cause steatosis, fibrosis, cirrhosis and hepatocellular carcinoma (HCC) and is a main indication for liver transplantation [2]. HCV is an enveloped virus that belongs to the *Hepacivirus* genus within the *Flaviviridae* family. The positive sense single-strand RNA genome encodes for a polyprotein that is cleaved by cellular and viral proteases into 10 viral proteins: three structural proteins (core, envelope proteins E1 and E2), the p7 polypeptide and six non-structural proteins (NS2, NS3, NS4A, NS4B, NS5A, NS5B). The structural proteins are main constituents of the virus particle whereas most of the non-structural proteins are required for RNA replication. Assembly of virus particles is tightly linked to cytosolic lipid droplets (LDs), where core accumulates, and components of the low density lipoprotein (LDL) pathway, most notably apolipoprotein E (reviewed in [3]).

As for all viruses, the HCV life cycle strongly depends on host cell factors promoting or restricting its replication. Thus, a tight interplay between viral and cellular proteins can be assumed to regulate virus replication and survival of the host cell. Numerous host cell factors involved in the HCV life cycle have been reported so far (reviewed in [4]), often based on RNA interference screens with genome-wide or more selective siRNA libraries [5]–[17]. However, the overlap between identified cellular factors is marginal, likely resulting from the use of different siRNA libraries and experimental conditions, but also from several technical limitations that are inherent to high-throughput siRNA screens [18]. Importantly, in most cases the specific roles of these factors for the HCV life cycle have not been clarified.

Heterogeneous nuclear ribonucleoprotein K (HNRNPK) is a polycytidine-binding protein originally identified as a component of the heterogeneous nuclear ribonucleoprotein complex [19]. HNRNPK is able to interact with RNA, DNA and multiple proteins and is involved in various cellular processes including chromatin remodeling, regulation of transcription, splicing and RNA translation [20] and was shown to assemble on DNA either as a transcriptional activator or as repressor [21], [22]. In addition, mRNA stability [23] as well as alternative splicing [24] can be affected by binding of HNRNPK to mRNA structures. The presence of multiple phosphorylation sites in HNRNPK suggests that this protein is also involved in several cellular signaling pathways [25].

Interestingly, HNRNPK has been reported to be involved in the life cycle of different viruses by either direct interaction with viral proteins [26], [27] or by affecting signal transduction and gene expression in a more indirect manner [28], [29]. However, the effect of HNRNPK as a pro- or anti-viral factor strongly differs between the various virus systems.

In the present study we conducted a siRNA-based screen and identified several cellular host factors affecting the HCV life cycle. This included HNRNPK that was found to selectively suppress production of infectious HCV particles. Our results suggest that HNRNPK might regulate the availability of viral RNA for the formation of infectious HCV particles.

## Results

### High-throughput siRNA screen identifies cellular factors involved in the HCV life cycle

**Figure 1:**
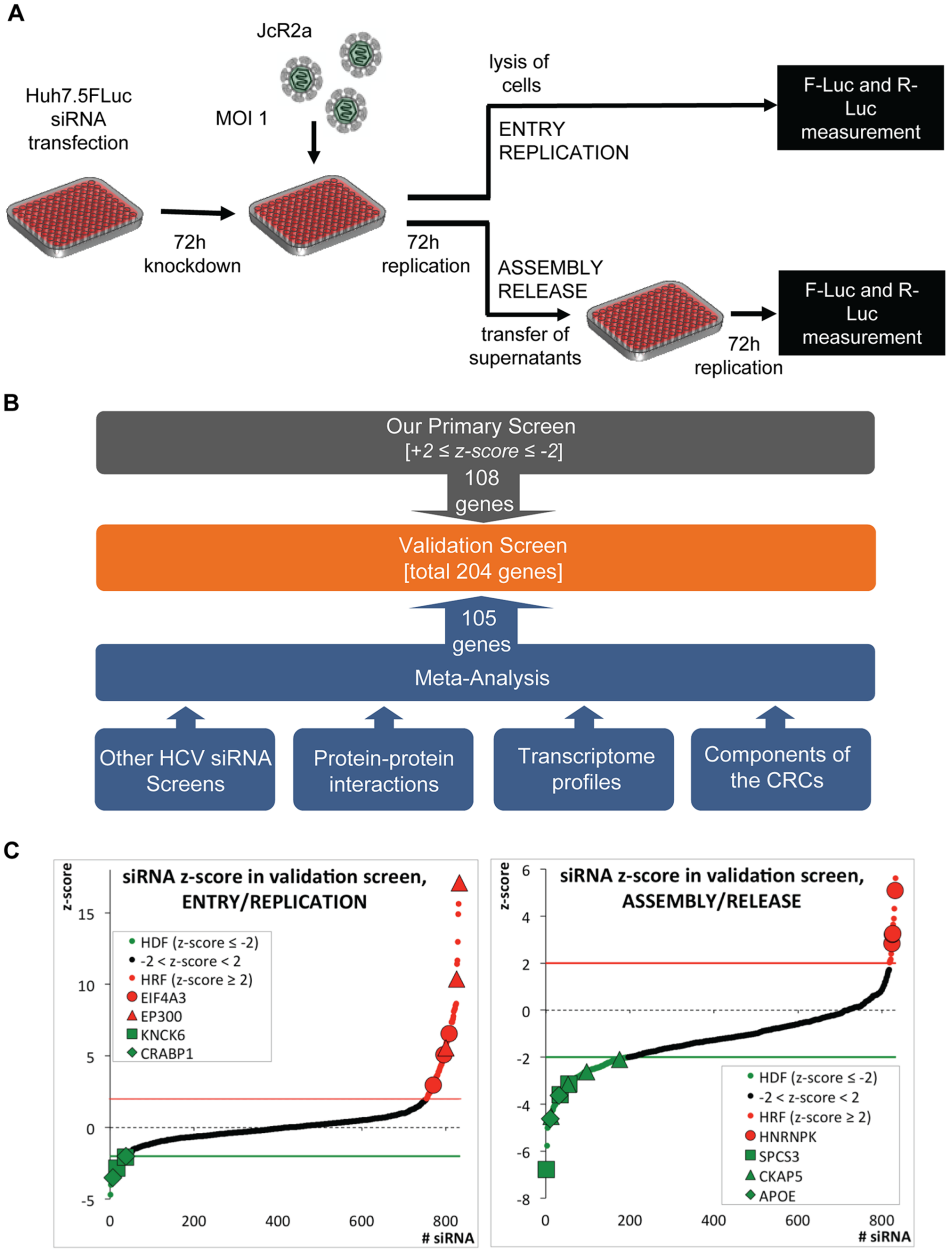
High-throughput siRNA screen used to identify cellular factors involved in the HCV life cycle. (A) Schematic of the screen. Primary and validation siRNA screen were performed as described in Supplemental Experimental Procedures. Huh7.5 cells stably expressing *Firefly* luciferase (FLuc) were infected with the *Renilla* luciferase HCV reporter virus JcR2a (MOI  = 1 TCID_50_/cell) 72 h after silencing. To determine the impact of knock-down on HCV entry and replication, cells were lysed 72 h after infection and *Renilla* luciferase activity was measured. To determine the impact of knock-down on virus production, naïve Huh7.5 FLuc cells were infected with supernatants of transfected cells and 72 h later, *Renilla* luciferase activity was determined. To account for potential cytotoxic effects of siRNAs FLuc was measured in the same lysates by using dual luciferase assay. (B) Approach used to define genes for the extended validation screen. Based on highest z-scores, 108 genes were selected from the primary siRNA screen (grey box). In addition, 105 genes were deduced from a meta-analysis (blue box) by using the indicated data sets (for further details see Experimental Procedures and S1B Table). To each of these data sets a weighting factor (wf) was given, reflecting their reliability and relevance. Nine of the genes selected by this meta-analysis were also identified as hits in the primary screen. Thus, a total 204 genes were used for the validation screen (orange box). (C) Results of the extended validation siRNA screen for entry/replication and assembly/release. The screen was performed in four replicates, using 4 different siRNAs per gene in a 96-well plate format. All replicates were used to compute z-scores for each siRNA (black dots). Hit siRNAs were defined by a z-score of ≤−2 (green line) in case of host dependency factors (HDF, green dots) or a z-score ≥+2 (red line) in case of host restriction factors (HRF, red dots). Only genes for which at least two siRNAs scored positive were considered. We identified 40 genes as HDFs and 16 genes as HRFs. Genes targeted by siRNAs that gave highly significant z-scores are indicated. EIF4A3, eukaryotic translation initiation factor 4A3; EP300, E1A binding protein p300; KCNK6, potassium channel, subfamily K, member 6; CRABP1, cellular retinoic acid binding protein 1; SPCS3, signal peptidase complex subunit 3 homolog; CKAP5, cytoskeleton associated protein 5; ApoE, apolipoprotein E.

In search for host cell factors involved in the HCV life cycle, a two-step high-throughput siRNA screen was conducted (Fig. 1A). We used the extended druggable genome siRNA library covering a total of 9,102 human genes with known or predicted functions and suitable as potential drug targets. The first part of the screen covered HCV entry and replication, whereas the second part covered production of infectious extracellular virus particles (assembly and release). For each gene, three different siRNAs were tested individually by using solid-phase reverse transfection. Huh7.5 FLuc cells, stably expressing the *Firefly* luciferase, were infected with the *Renilla* luciferase reporter virus JcR2a [9] that was derived from the highly assembly competent HCV chimera Jc1 [30]. Seventy-two hours after infection, cells were harvested to measure virus replication by *Renilla* luciferase assay, thus determining the impact of knock-down on HCV entry and replication. *Firefly* luciferase activity was quantified to exclude false-positive hits caused by knock-down-mediated alteration of cell growth and viability. To monitor the impact of knock-down on production of extracellular virus, culture supernatants of siRNA-transfected cells were used to inoculate naïve Huh7.5 FLuc cells and 72 h later, HCV replication was determined by *Renilla* luciferase assay; *Firefly* luciferase activity was also determined to exclude cell growth effects caused by the inoculum.

The primary siRNA screen was performed in three replicates and identified in total 78 host dependency factors (HDFs) and 29 host restriction factors (HRFs) (S1A Fig.; S1A Table). Gene Ontology (GO) (Gene Ontology Consortium, 2010) of candidate host factors revealed the biological processes implicated in the HCV life cycle: transport, transcription and transcription regulation (S1B Fig. and S1C, D Table).

Given the technical limitations that are inherent to such high-content siRNA-based screens, we attempted to increase comprehensiveness and reliability of our screen by conducting a meta-analysis that was based on the following data sets (Fig. 1B and S1B Table): (i) our own and published HCV siRNA screens with genome-wide or selected siRNA libraries [5]–[10], [12]–[16], [31]–[37]; (ii) a genome-wide high-throughput yeast-two hybrid protein interaction study [38]; (iii) a comparative analysis of the proteome of crude HCV replication complexes (CRCs) conducted by us and others [39]; (iv) a comparative transcriptome analysis between low- and highly permissive Huh-7 cells [40], [41], because the degree of permissiveness might be due to expression levels of dependency and restriction factors; (v) a comparison of a mouse and a human hepatocytic cell line (Hep56.1D and HuH6, respectively) with or without a subgenomic HCV replicon [42], [43], assuming that differences in expression levels of dependency and restriction factors might be more accentuated in cells that are much less permissive for HCV as compared to Huh-7. In this way, a total of 204 genes (108 from our primary screen and 96 deduced from the meta-analysis) were tested in an extended validation screen for their impact on the HCV life cycle.

For the validation screen, we used 4 different siRNAs per gene purchased from a different supplier and the same set-up applied for the primary screen (Fig. 1A). Hits were defined by a z-score of ≤−2 for HDFs or ≥+2 for HRFs, achieved with at least two different siRNAs per gene in 4 independent repetitions. Fig. 1C summarizes mean z-scores of all individual siRNAs, combining the four replicates, sorted from the lowest HDFs to the highest HRFs. In total, we were able to validate 40 HDFs and 16 HRFs affecting the HCV life cycle (S1A Table). This corresponds to 21% of the hits identified in the primary screen and 28% of the candidates selected by our meta-analysis.

To reveal cellular processes critically involved in the HCV life cycle, we performed an integrative computational analysis (S2 Fig.; S1C, D, F Table). Consistent with earlier reports, we identified enrichments of, amongst others, intracellular protein transport pathways such as the COP-I system [6], factors involved in the epidermal growth factor receptor signaling pathway [44], signal recognition particle receptor-dependent transport and signal peptide processing (reviewed in [45]) or the low-density-lipoprotein-pathway, consistent with the tight link of HCV assembly with intracellular lipid synthesis and storage systems [46].

### Selection of HNRNPK for mechanistic studies

**Figure 2:**
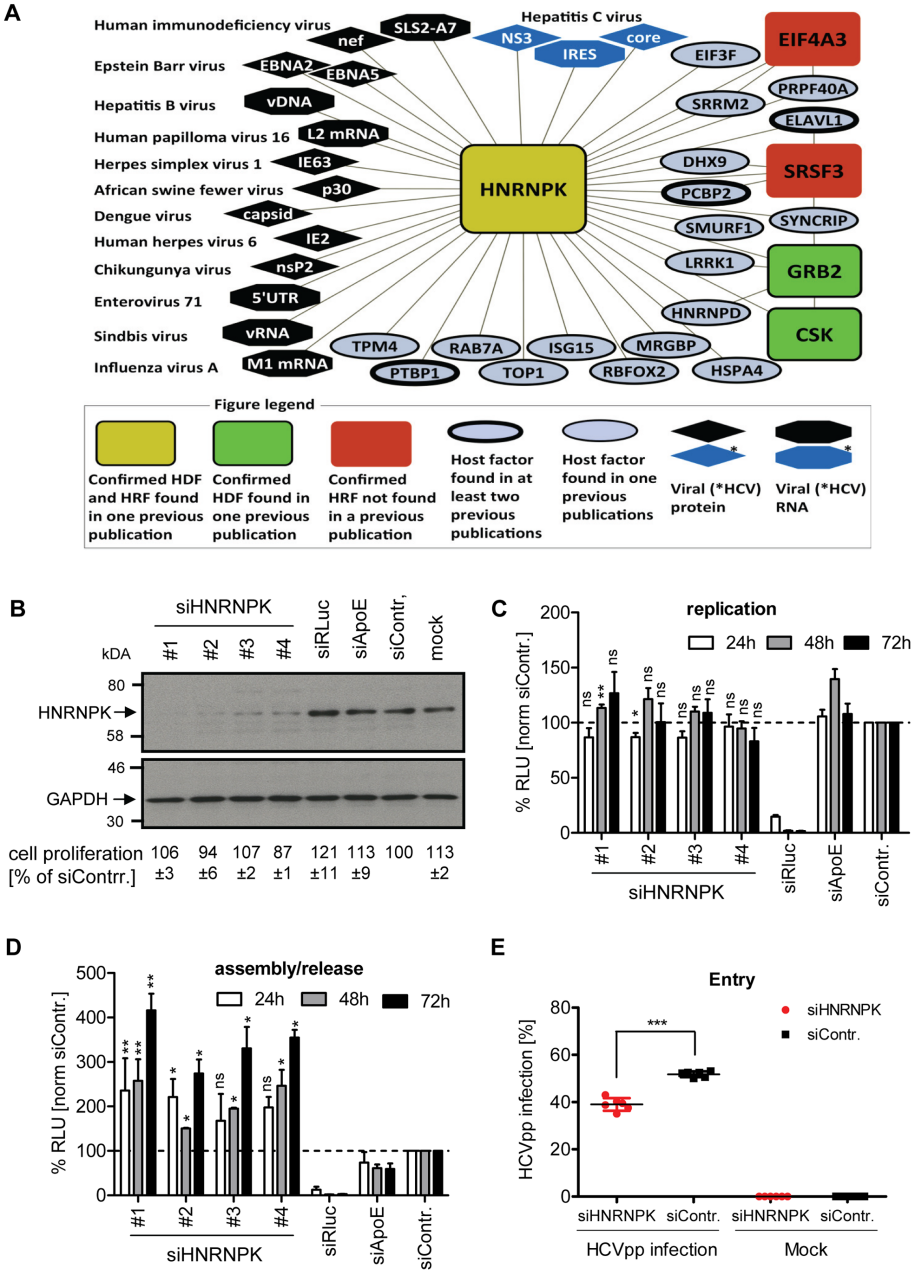
HNRNPK restricts HCV assembly/release but does not affect viral RNA replication. (A) HNRNPK – virus interaction network. The network shows all human cell proteins (ovals and rectangles), viral proteins (diamonds) and viral RNAs (octagons) and reported interactions with HNRNPK. (B) Silencing efficiency of HNRNPK siRNAs. Huh7.5 cells were electroporated with 2.5 µM of indicated siRNA or mock electroporated 48 h prior to infection with the *Renilla* luciferase reporter virus JcR2a (MOI  = 0.4 TCID_50_/cell). Cells were harvested 48 h later. Western blot was performed to determine silencing efficiency achieved with siRNAs specified in the top. HNRNPK was detected by using a mono-specific antibody; GAPDH served as loading control. siRNA targeting *Renilla* luciferase served as positive control for entry/replication assay, siRNA targeting ApoE was used as positive control for assembly/release assay, non-targeting siRNA (siContr.) and mock-treated cells were used as a negative controls. Western Blot represents one of two independent experiments. For HNRNPK silencing, the same siRNA sequences were used as in the validation screen. Quantification of cytotoxicity assays (cell proliferation) using cells transfected with given siRNAs are specified below each lane. Given are mean values ±SD of two independent experiments, normalized to siContr.-silenced cells. (**C**) Effect of HNRNPK silencing on HCV RNA replication or (D) virus particle production. Huh7-Lunet cells were co-electroporated with 2.5 µM of indicated siRNA and JcR2a in vitro transcripts. Non-targeting siRNA was used as negative control (siContr.). Cells and virus-containing supernatants were harvested at given time points after electroporation. (C) Cell lysates were used for measuring *Renilla* luciferase activity (relative light units, RLU) to detect effects on HCV replication. (D) Virus-containing supernatants of cells harvested at given time points were used for infection of naïve Huh7.5 cells and *Renilla* luciferase activity was determined 72 h later to estimate effects of HNRNPK silencing on HCV particle production. All values were normalized to cell viability. Non-targeting control siRNA (siContr.) was set to 100% (dotted line). Bars represent the mean ±SD of three independent experiments. (E) Effect of HNRNPK silencing on HCV entry. Huh7.5 cells were electroporated with 2.5 µM of a mixture of siRNA #1 - #4 targeting HNRNPK or the non-targeting siRNA (siContr.). After 48 h, cells were transduced with YFP-HCVpp (Con1) and the number of infected cells was determined by flow cytometry 72 h later. Non-transduced cells served as negative control. ***, P-value ≤0.0005; **, P-value ≤0.005; *, P-value ≤0.05; ns, non-significant. Statistical analysis was performed by using Student's t-test, referred to the non-targeting control siRNA (siContr.).

One of the most striking phenotypes was obtained upon knock-down of heterogeneous nuclear ribonucleoprotein K (HNRNPK), which had an opposing effect on both analyzed steps of the HCV life cycle. While knock-down of HNRNPK expression reduced virus entry/replication, albeit to a very moderate extent, virus assembly/release was profoundly enhanced in case of knock-down with 5 of the 7 selected siRNAs (S1A Table). Moreover, HNRNPK was not only found in our meta-analysis, but also reported in earlier studies as HCV-modulating host cell factor [5], [17], [38], arguing that HNRNPK might play an important role for the viral life cycle (S1B Table). In fact, HNRNPK was reported to interact with HCV core [47], NS3 [38] and the internal ribosome entry site (IRES) residing in the 5′ non-translated region of the viral genome [48]. Moreover, HNRNPK has also been reported to be involved in the life cycle of many other viruses such as Chikungunya virus [49], Influenza A virus [50] or Sindbis virus [26], interacting with viral proteins or nucleic acid structures (Fig. 2A). Thus, given the consistency and robustness of results we obtained for HNRNPK and HCV, the opposing phenotype on entry/replication versus assembly/release and the unknown mechanism by which this host cell factor affects the HCV life cycle, we selected HNRNPK for further characterization.

### HNRNPK suppresses production of infectious HCV but not dengue virus particles

In the first set of experiments we determined whether HNRNPK expression would be affected by HCV infection. However, neither at the level of mRNA, nor with respect to HNRNPK abundance we observed alterations in HCV-infected cells as compared to control cells (S3A and B Fig., respectively).

With the aim to separate the effect of HNRNPK on HCV assembly/release from its contribution to viral RNA replication, we co-electroporated HNRNPK-specific siRNAs, together with the JcR2a RNA genome, into Huh7-Lunet cells. These cells are highly permissive for viral replication as well as assembly and release, but support HCV entry only poorly due to low amounts of CD81 [51]. Thus, an apparent increase of viral replication due to virus spread could be excluded with these cells. Western Blot revealed efficient knock-down by each of the four siRNAs (Fig. 2B). In contrast, non-targeting control siRNA (siContr.) as well as siRNAs targeting either the luciferase sequence within the genome of JcR2a or the assembly factor apolipoprotein E (ApoE) did not affect HNRNPK abundance. None of these siRNAs exerted cytotoxicity (Fig. 2B). As shown in Fig. 2C, depletion of HNRNPK had no significant effect on viral replication whereas the positive control targeting the *Renilla* luciferase sequence in the viral genome (siRLuc) completely abolished replication. Importantly, amounts of infectious extracellular virus produced by HNRNPK-depleted cells revealed a ∼4-fold increase as compared to siContr.-transfected cells, corroborating the suppression of HCV production by HNRNPK (Fig. 2D). Note that the conditions used here were optimized for HNRNPK-, but not ApoE-specific knock-down for which an shorter incubation period leads to higher silencing efficiency [52]. Therefore, reductions of virus titers achieved in the latter case were rather moderate.

Although HNRNPK knock-down exerted a predominant effect on HCV particle production, in the primary screen it appeared as a dependency factor and reduced HCV entry/replication. Since an effect on RNA replication had been excluded, we determined the possible role of HNRNPK for virus entry by using HCV pseudoparticles (HCVpp) (Fig. 2E). Therefore, we silenced HNRNPK expression for 48 h by using a mix of the 4 different siRNAs and infected these cells with a YFP gene-transducing HCVpp using Con1- (gt1b) derived envelope glycoproteins. After 72 h the number of infected cells was determined by flow cytometry. Consistent with the primary screen data we found that HNRNPK knock-down caused a slight, but statistically significant reduction of the number of infected cells as compared to cells transfected with the non-targeting control siRNA (siContr.). Thus, HNRNPK appears to play a role in the early steps of the HCV replication cycle. However, given the more striking effect of HNRNPK knock-down on the production of infectious HCV particles, we focused our further analysis on this aspect.

**Figure 3:**
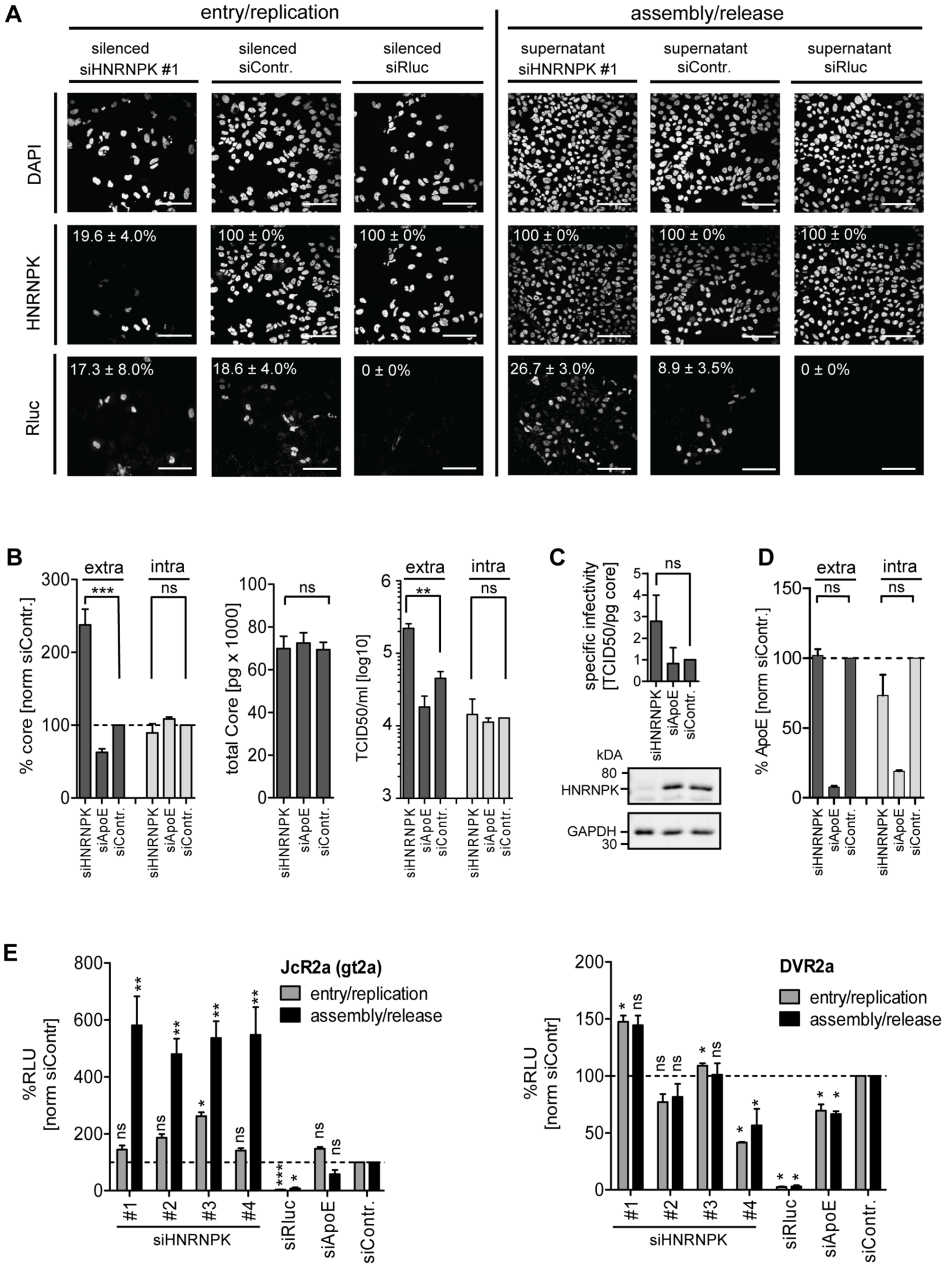
HNRNPK primarily affects HCV assembly. (A) Knock-down efficiency of HNRNPK as determined by immunofluorescence analysis. Huh7.5 cells were electroporated with 2.5 µM of siRNA and 48 h later infected with JcR2a (MOI  = 0.4 TCID_50_/ml). For monitoring the effect of silencing on HCV entry/replication, cells were fixed 48 h post infection (left panel). Supernatants of transfected cells were used to inoculate naïve Huh7.5 cells. Seventy two hours later, cells were fixed and analyzed to detect an effect of HNRNPK silencing on assembly/release (right panel). To monitor knock-down efficiency and HCV-replication/spread cells were stained for HNRNPK or *Renilla* luciferase (R-Luc), respectively, as indicated in the left of each panel. Nuclear DNA was stained with DAPI. Percentage of silenced and infected cells and standard deviations were calculated by counting at least 300 individual cells. Images were acquired with a confocal microscope; scale bars refer to 100 µm. (B - D) Silencing of HNRNPK expression enhances extracellular amounts of core and infectivity, but does not affect ApoE secretion. Huh7.5 cells were co-electroporated with 2.5 µM of siRNA specified in the bottom and 5 µg of Jc1 RNA. ApoE-specific siRNA was used as a control. (B) Left two panels: extra- and intracellular core amounts as well as total core amounts were measured 72 h post electroporation, respectively. Right panel: virus titer as determined by limiting dilution assay. (C) HNRNPK does not affect specific infectivity as determined by calculating the ratio of TCID_50_ titer and core amount. Knock-down efficiency was determined by Western blot (lower panels). GAPDH served as loading control. (D) Knock-down of HNRNPK expression does not affect secretion of ApoE. Cells were transfected with siRNAs specified in the bottom and amounts of ApoE contained in culture supernatant or within cells were measured 72 h later by ELISA. (E) Restriction of assembly/release by HNRNPK is specific to HCV and not found with DENV (left and right panel, respectively). Forty-eight hours post silencing, siRNA-transfected cells were infected with HCV (JcR2a reporter virus; MOI  = 0.4 TCID_50_/ml; left panel) or DENV (DVR2a reporter virus; MOI  = 1 TCID_50_/ml; right panel). To determine the impact of knock-down on viral entry/replication (grey bars), cells were lysed 48 h post infection. To measure knock-down impact on virus production (black bars), supernatants of transfected cells were used to inoculate Huh7.5 cells that were lysed 72 h later. Virus replication was quantified by measuring *Renilla* luciferase activity (relative light units, RLU) and values were normalized to cell viability. Non-targeting control siRNA (siContr.) was set to 100% (dotted line). SiRNAs targeting the *Renilla* luciferase sequence in the reporter virus genomes served as positive control. Bars in all panels represent the mean ±SD of at least two independent experiments. ***, P-value ≤0.0005; **, P-value ≤0.005; *, P-value ≤0.05; ns, non-significant. Statistical analysis was performed by using Student's t-test, referred to the non-targeting control siRNA (siContr.).

To corroborate our results with respect to the role of HNRNPK for HCV assembly/release, we conducted an immunofluorescence analysis of JcR2a-infected cells (Fig. 3A). HNRNPK depletion did not affect HCV replication (as determined by abundance of the *Renilla* luciferase protein encoded in the reporter virus genome) at the single cell level (Fig. 3A, left panel). In contrast, amount of infectious virus contained in the supernatant of HNRNPK-silenced cells was strongly increased, as reflected by a higher number of infected cells (Fig. 3A, right panel). To exclude an effect of HNRNPK knock-down on *Renilla* luciferase and to determine whether HNRNPK enhanced virus production or particle infectivity, we co-transfected Huh7.5 cells with siRNA and a reporter-free Jc1 genome and determined intra- and extracellular core protein amounts along with infectious virus titers (Fig. 3B). While amounts of core protein and infectious virus particles in transfected cells were unaltered by HNRNPK depletion, amounts of core and infectious virus particles in culture supernatants were clearly elevated (Fig. 3B). However, total amount of core did not change, because intracellular core level was ∼10-fold higher and therefore, changes in amounts of secreted core protein caused only minor changes in total core protein abundance. Although there was a trend towards slightly increased specific infectivity of virus particles released from HNRNPK knock-down cells, the difference to control cells was not statistically significant suggesting that HNRNPK primarily contributes to virus production (Fig. 3C). The fact that extracellular infectivity titers were elevated without concomitant reduction of intracellular infectivity titers argued against a pure virus release phenotype of HNRNPK knock-down. Moreover, intra- and extracelluar amounts of ApoE were not altered in HNRNPK knock-down cells (Fig. 3D), excluding a general effect of HNRNPK depletion on the secretory pathway. Finally, we found that HNRNPK depletion elevated virus production independent from the analyzed genotype. By using chimeric genomes encoding for gt1a (H77) or gt1b (Con1) structural proteins we observed an increase of HCV assembly/release that was well comparable to the one detected with the gt2a chimera JcR2a (S4A – C Fig.). Moreover, HNRNPK-dependent suppression of virus production was independent from the assembly competence of the used virus genome; elevated virus production upon HNRNPK knock-down was also observed with the original JFH-1 isolate that supports assembly only poorly as compared to the highly assembly-competent variant Jc1 [30] (S4D Fig.).

Given the close relationship between HCV and Dengue virus (DENV) that both belong to the family *Flaviviridae* and the role of HNRNPK in the life cycle of several other viruses (Fig. 2A), we determined whether HNRNPK also affects production of infectious DENV particles. To this end, we utilized a DENV *Renilla* luciferase reporter virus that was tested in parallel to the HCV reporter virus JcR2a. In agreement with results described above, we detected profound enhancement of HCV assembly/release with no overt effect on RNA replication (Fig. 3E, left panel). In contrast, HNRNPK knock-down had no impact on production of infectious DENV (Fig. 3E, right panel). In summary, these results suggest that HNRNPK enhances production of infectious HCV particles, rather than particle release, and this effect appears to be specific for HCV.

### RNA- and protein-binding domains of HNRNPK are essential for suppression of HCV particle production

**Figure 4:**
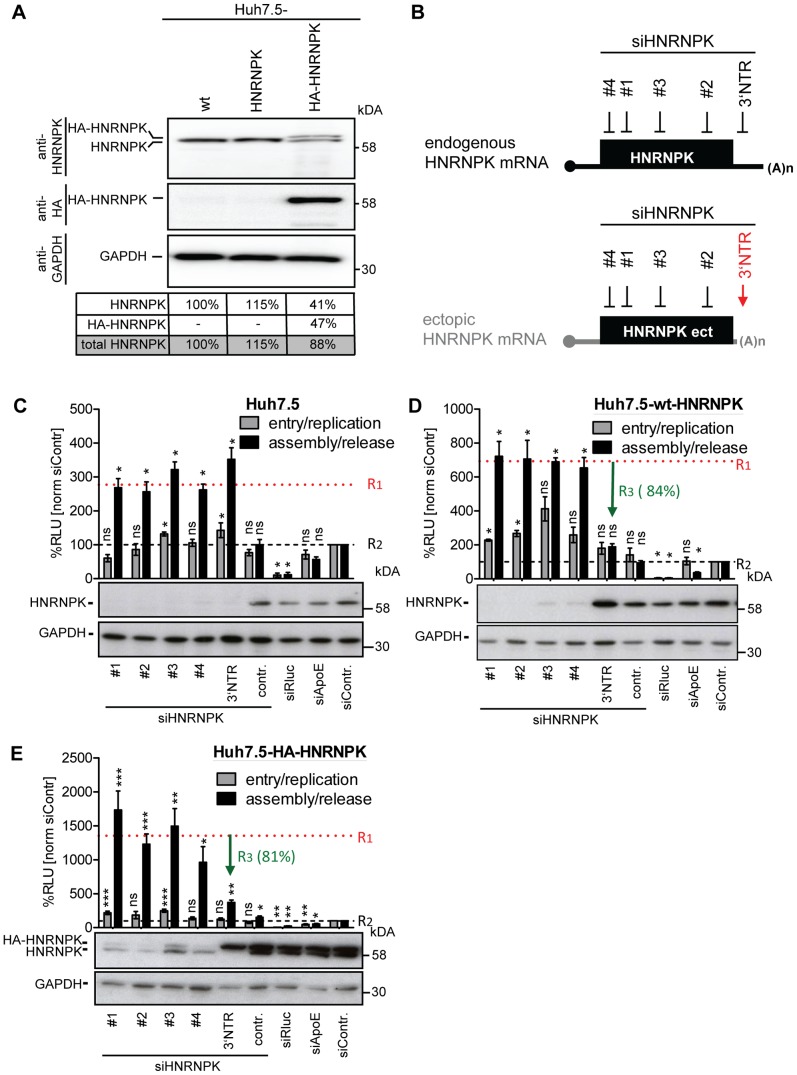
Ectopic expression of HNRNPK restores suppression of virus production in HNRNPK-silenced cells. (A) Abundance of endogenous HNRNPK drops upon expression of ectopic HNRNPK. Expression levels of HNRNPK in wild type Huh7.5 cells (wt) or Huh7.5 cells additionally expressing the untagged (HNRNPK) or HA-tagged HNRNPK (HA-HNRNPK) were determined by Western blot. Amounts of endogenous or ectopic HNRNPK were quantified and total amounts of HNRNPK were calculated for each condition. (B) Location of siRNA target sites in endogenous and ectopically expressed HNRNPK mRNA. Targeting sites of individual HNRNPK siRNAs (#1 to #4) reside in the coding region of both mRNAs. The targeting site of siHNRNPK-3′NTR (red arrow) matches only to the 3′NTR of the endogenous HNRNPK mRNA and therefore does not result in a knockdown of ectopically expressed HNRNPK. (C - E) Restoration of assembly/release restriction in HNRNPK-silenced cells. (C) Naïve Huh7.5 cells [Huh7.5], (D) Huh7.5 cells stably expressing the exogenous HNRNPK [Huh7.5-wt-HNRNPK] or (E) a HA-tagged HNRNPK [Huh7.5-HA-HNRNPK] were electroporated with 2.5 µg of the indicated siRNA. Cells were infected with JcR2a 48 h after silencing (MOI  = 0.4 TCID_50_/ml). To monitor viral entry/replication (grey bars) cells were lysed 48 h post infection and *Renilla* luciferase activity was measured. To determine virus production (assembly/release), supernatants of transfected cells were used to inoculate Huh7.5 cells (black bars) and 72 h later *Renilla* luciferase activities were measured (relative light units, RLU). Values were normalized to cell viability and the non-targeting control siRNA (siContr.) that was set to 100% (black dotted line). Silencing efficiency was determined by HNRNPK-specific Western blot. GAPDH was used as loading control. Bars represent the mean ±SD of four independent experiments. To quantify suppression of virus production by ectopic HNRNPK expression, mean values obtained with siRNA HNRNPK#1 to #4 (dotted horizontal red line; value R1) and siHNRNPK-3′NTR targeting only endogenous HNRNPK were compared with each other (value R3). Values were normalized to the ones obtained with the siContr. that was set 100% (value R2; dotted horizontal black line). Thus, R3 values reflect the relative suppression of HCV particle production achieved with ectopically expressed HNRNPK. ***, P-value ≤0.0005; **, P-value ≤0.005; *, P-value ≤0.05; ns, non-significant. Statistical analysis was performed by using Student's t-test, referred to the non-targeting control siRNA (siContr.).

To exclude undesired off-target effects caused by the used siRNAs and to set up an assay allowing mapping studies of HNRNPK domains of relevance for HCV assembly, we tried to overexpress HNRNPK by lentiviral transduction. In the course of these experiments we noted that abundance of endogenous HNRNPK dropped upon expression of ectopic HNRNPK. As shown in Fig. 4A, cells expressing ectopically either wild type or HA-tagged HNRNPK did not contain higher amounts of total HNRNPK than control cells only expressing endogenous HNRNPK, arguing that expression level of this protein is tightly regulated. Due to this tight regulation of HNRNPK expression, we were not able to explore whether overexpression would result in a stronger suppression of HCV virus production.

To overcome this limitation, we aimed to restore HNRNPK-mediated suppression of HCV particle production in knock-down cells by ectopic expression of a siRNA-resistant variant. Huh7.5 cell pools stably expressing either wild type or HA-tagged HNRNPK (Huh7.5-wt-HNRNPK and Huh7.5-HA-HNRNPK, respectively), in addition to residual amounts of endogenous HNRNPK, were established. The ectopically expressed HNRNPK genes lacked the authentic 3′ NTR and thus, were resistant to the siRNA targeting the 3′ end of the endogenous HNRNPK mRNA (si-3′NTR), but sensitive to siRNAs #1 - #4 targeting sequences in the coding region (Fig. 4B). To demonstrate comparable knock-down efficiency of all 5 siRNAs, naïve Huh7.5 cells were transfected with each siRNA individually and infected with JcR2a (Fig. 4C). Virus amounts contained in culture supernatants were determined by infection of naïve Huh7.5 cells, *Renilla* luciferase activity was quantified 72 h later and normalized to cell viability and the non-targeting control siRNA (siContr.). A siRNA with a non-functional HNRNPK recognition site served as additional control (siHNRNPKcontr.). All HNRNPK-specific siRNAs reduced expression of the target gene, concomitant with an increase of amounts of infectious extracellular HCV (Fig. 4C). Importantly, HNRNPK silencing with siRNAs #1 - #4 depleted both endogenous and ectopic HNRNPK, whereas the siRNA targeting the 3′ NTR depleted only endogenous HNRNPK (Fig. 4D, E). Although the absolute values of assembly/release enhancement caused by HNRNPK knock-down differed between individual experiments and cell pools, the relative capacity of ectopically expressed HNRNPK to suppress HCV particle production (termed R3 value) was well comparable between cells expressing different siRNA-resistant genes (green arrow in Fig. 4D, E). These results unequivocally confirmed specificity of the knock-down phenotype and they showed that HA-tagged HNRNPK is fully functional (Fig. 4E).

**Figure 5:**
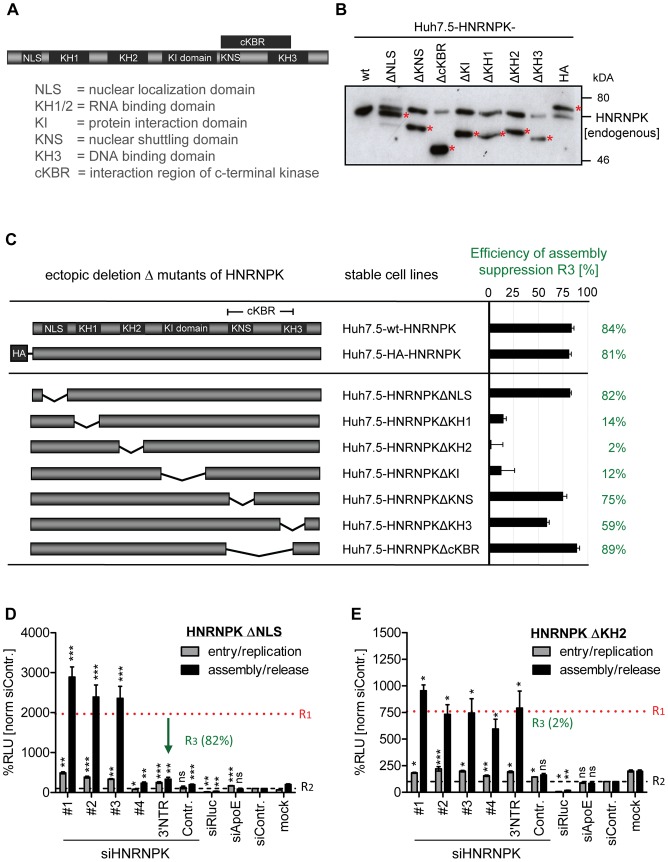
RNA and protein binding domains of HNRNPK are essential for restriction of HCV particle production. (A) Schematic of HNRNPK protein domains: NLS [aa 21–37]; KH1 [aa 46–98]; KH2 [aa 149–197]; KH3 [aa 391–439]); KI [aa 246–337]; cKBR [aa 338–425]; KNS [aa 338–361]. (B) Stable expression of HNRNPK mutants in Huh7.5 cells. Expression levels of ectopically expressed HNRNPK proteins (labeled with red asterisks), in addition to the endogenous protein (wt), were determined by Western blot. (C) Suppression of HCV particle production by HNRNPK mutants. Huh7.5 cell pools stably expressing HNRNPK proteins specified in the middle column were electroporated with 2.5 µg of siHNRNPK-3′NTR targeting only endogenous HNRNPK. Forty eight hours later, cells were infected with JcR2a (MOI  = 0.4 TCID_50_/ml). R3 values representing the capacity of a given HNRNPK mutant to suppress HCV particle production were calculated as described in the legend to Fig. 4. Examples of results are shown in panel (D) for HNRNPKΔNLS and (E) for HNRNPKΔKH2. Note that the binding region for siHNRNPK#4 is lost because of the NLS deletion. The complete data sets are displayed in S5 Fig. ***, P-value ≤0.0005; **, P-value ≤0.005; *, P-value ≤0.05; ns, non-significant. Statistical analysis was performed by using Student's t-test, referred to the non-targeting control siRNA (siContr.).

Taking advantage of this knock-down/rescue assay, we next mapped HNRNPK domains that are of relevance for HCV particle production. HNRNPK is a multifunctional protein composed of different domains [20] (Fig. 5A) including a predicted nuclear localization signal (NLS) and a nuclear shuttling domain (KNS), KH1 and KH2 domains capable of binding single strand RNA, a KH3 domain binding to DNA, a KI region responsible for protein-protein interaction and an interaction region with c-terminal kinase (cKBR) [20]. For mapping studies we generated a series of HNRNPK mutants lacking each of these domains individually (Fig. 5B). These variants were tested for their capacity to restore suppression of HCV particle production in cells with knock-down of endogenous HNRNPK. As shown in Fig. 5C, HNRNPK mutants lacking the NLS, the cKBR domain or the KNS were only moderately affected in their capability to suppress HCV particle production. For these mutants, R3 values ranged between 89% and 75% (Fig. 5C and D; for complete data set see S5 Fig.), showing that the deleted domains are not essential for HNRNPK-mediated suppression of HCV particle production. While deletion of the DNA binding domain (KH3) resulted in an intermediate phenotype (R3  = 59%; Fig. 5C and S5E Fig.), removal of either one of the two RNA binding domains (KH1 and KH2) or the protein binding domain (KI) rendered the protein virtually inactive (Fig. 5C, E and S5D, F Fig.). These results argue for an essential role of the RNA-binding KH-domains and the protein-protein interaction domain in limiting HCV particle production, whereas the other domains of HNRNPK appear to be dispensable.

### Interaction of HNRNPK with HCV RNA correlates with suppression of virus production

HNRNPK has been reported to be involved in the replication of multiple viruses and in some cases, regulatory functions of HNRNPK are based on its interaction with viral proteins (Fig. 2A). With respect to HCV, an interaction between HNRNPK and full length or truncated core as well as NS3 has been described [38], [47]. However, these interaction studies have been conducted with artificial expression systems, but the relevance in more authentic infection-based systems has not been addressed. Therefore, we investigated HNRNPK interaction with core and NS3 using either wild type or HA-tagged HNRNPK-expressing cells containing a subgenomic replicon or a full length HCV genome (S6 Fig.). HA-specific immunoprecipitation assays confirmed that HA-HNRNPK coprecipitated with core and NS3, although efficiency appeared rather low (∼1% of the respective intracellular HCV protein). Nevertheless, specificity of the pull-down was confirmed by lack of HNRNPK coprecipitation with NS5A or GAPDH and the absence of core and NS3 in immunoprecipitations with non-tagged HNRNPK (S6 Fig.). Moreover, we found that mutants unable to suppress HCV assembly were also impaired in interaction with core protein as well as NS3 whereas a HNRNPK mutant capable to suppress virus particle production still interacted with these two viral proteins with efficiency comparable to the HA-tagged wild type (S7 Fig.).

**Figure 6:**
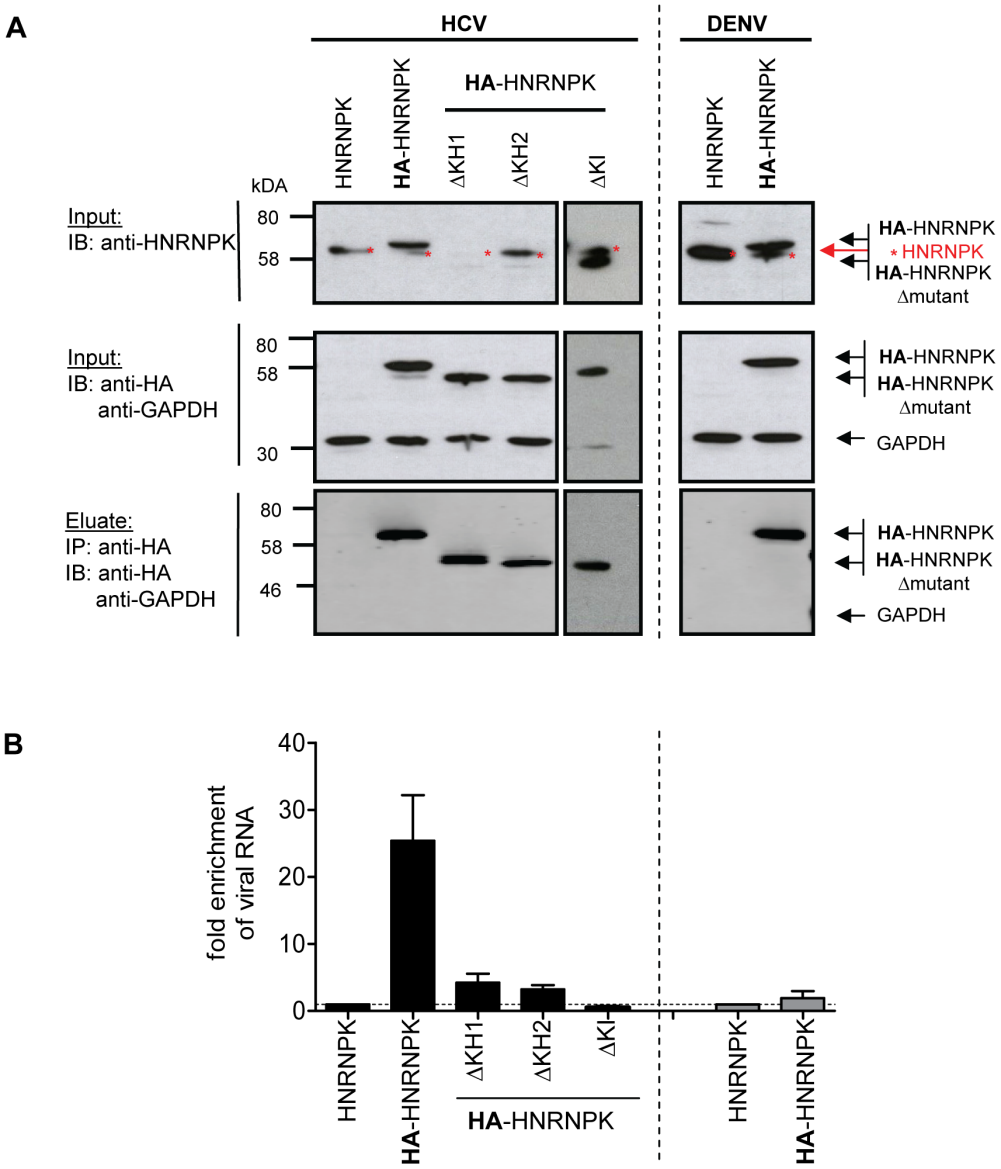
HNRNPK interacts with HCV but not DENV RNA genomes. (A) Immunoprecipitation of HA-HNRNPK. Huh7.5 cells stably expressing unaltered, HA-tagged HNRNPK, or HA-tagged mutants ΔKH1, ΔKH2 or ΔKI were electroporated with genomic Jc1 (HCV) RNA. Genomic Dengue Virus (DENV) RNA was used as a control. Cells were harvested 48 h post electroporation, lysates were used for HA-specific immunoprecipitation and captured complexes were analyzed by Western blot. Input was analyzed with a HNRNPK and a HA-specific antibody (upper and middle panel, respectively); GAPDH served as loading control. Immunocomplexes (corresponding to 10-fold the amount of the input) were analyzed with the HA-specific antibody (lower panel). Note that the ΔKH1 mutant is not recognized by the HNRNPK-specific antibody, but reacted with the HA-specific antibody. (B) Quantitative analysis of co-precipitated HCV and DENV RNA. Co-precipitated viral RNA was determined by RT-qPCR and normalized to input RNA. Fold enrichment of captured viral RNA was calculated by comparing the HA-tagged to the untagged HNRNPK-fraction (set to 1; dotted horizontal line). Bars represent the mean ±SD of at least three independent experiments.

To determine whether HNRNPK also interacts with HCV RNA, we conducted an analogous experiment, but immunocomplexes were analyzed for captured viral RNA using RT-qPCR, whereas efficiency of pull-down was determined by Western blot using HNRNPK- or HA-specific antibodies (Fig. 6). HNRNPK deletion mutants ΔKH1, ΔKH2 and ΔKHI were included, because of their inability to suppress HCV particle production. Cells expressing the non-tagged (wild type) HNRNPK served as technical control to determine background binding of HCV RNA to HA-specific beads. Finally, we included cells transfected with a fully functional DENV genome to determine whether observed phenotypes are specific to HCV. All HNRNPK constructs were well expressed and HA-tagged proteins were efficiently captured with high specificity (Fig. 6A). Analysis of immunocomplexes with an HCV- or DENV-specific RT-qPCR revealed specific co-capture of the subgenomic and the genomic HCV RNA with HA-HNRNPK whereas no such interaction was detected in case of DENV (Fig. 6B). Pull-down efficiency ranged between 10% and 25% of detected intracellular HCV RNA. Moreover, HNRNPK deletion mutants unable to restrict HCV particle production did not interact with viral RNA. Importantly, loss of interaction with HCV RNA was also found with mutant ΔKI lacking the protein-protein interaction domain and unable to suppress HCV particle production (Fig. 6 and 5, respectively). Taken together, the correlation between suppression of HCV production by HNRNPK and its interaction with viral RNA argues for a link between these two processes. The observation that HNRNPK mutants unable to interact with core and NS3 only weakly coprecipitated HCV RNA argues for an indirect interaction. Inhibition of virus production by HNRNPK was specific to HCV and not found with the related DENV for which no interaction of HNRNPK with viral RNA could be found (Fig. 6).

### Impact of HCV on HNRNPK expression and subcellular localization

**Figure 7:**
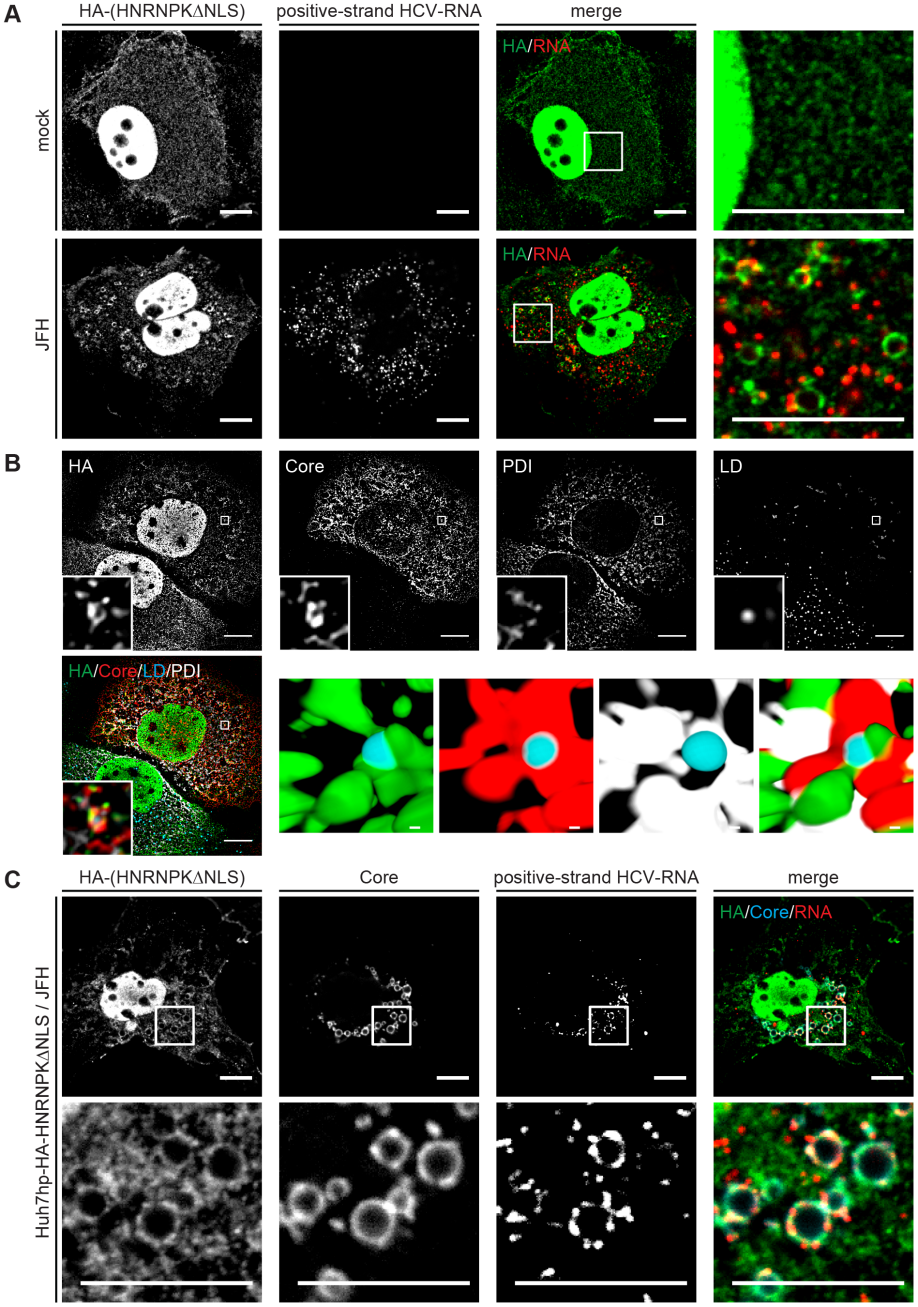
Redistribution of HNRNPK subcellular localization in HCV-containing cells and colocalization with viral RNA and core. (A) Subcellular localization of HNRNPK is altered during HCV infection. Huh7hp cells stably expressing the HA-tagged HNRNPKΔNLS mutant were electroporated with genomic JFH1 (HCV) RNA or mock-transfected and fixed 72 h later. Ha-tagged HNRNPKΔNLS was visualized by immunofluorescence staining using a HA-specific antibody whereas HCV positive-strand RNA was detected by FISH using the QuantiGene ViewRNA ISH Cell Assay (Affymetrix). Enlargements of the sections indicated by white squares in the merged images are shown in the corresponding right panels. Note the pronounced punctuated staining pattern in the HCV-containing cell. (B) Ha-tagged HNRNPKΔNLS and core were visualized as described above and ER and LDs were detected by using PDI-specific antibody and HCS LipidTOX Deep Red, respectively. The small inserts in the bottom of each panel represent 3D reconstructed enlargements of each corresponding image created with the Imaris 7.7.2 software package. Scale bars represent 10 µm or 1 µm in regular and magnified images, respectively. (C) Colocalization of HNRNPK with HCV core and RNA. Huh7hp cells stably expressing the HA-tagged HNRNPKΔNLS mutant were electroporated with HCV RNA as described above. Ha-tagged HNRNPKΔNLS, core and HCV positive-strand RNA were visualized as described above. Enlargements of the sections indicated by white squares are shown in the corresponding lower panels. Images in panels B and C were acquired with a confocal microscope; scale bars refer to 10 µm.

HCV assembly occurs in close proximity of LDs [53] where viral proteins, most notably core and NS5A accumulate [54]. Given the role of HNRNPK in HCV particle production and the interaction of this host cell factor with viral RNA we first determined whether subcellular localization of HNRNPK would be affected by HCV infection. To this end we purified LDs from HCV-infected cells by floatation gradient. Although we observed a strong enrichment of core protein in case of LDs isolated from HCV-infected cells, as well as copurification of the core-interacting protein DDX3 that served as positive control [55], HNRNPK was not visible in LD fractions (S8 Fig.). While this result suggested that HNRNPK is not recruited to the surface of LDs, we wondered whether subcellular distribution of HNRNPK might be affected by HCV infection. To address this question we conducted immunofluorescence studies comparing HCV-infected cells with control cells. Since abundance of HNRNPK in the cytoplasm is very low, we took advantage of the HA-tagged HNRNPK mutant lacking the predicted NLS. This mutant suppressed HCV particle production as efficiently as wild type HNRNPK and thus, was fully functional with respect to the studied phenotype (Fig. 5C, D). Although removal of the NLS increased abundance of HNRNPK in the cytoplasm, a substantial proportion of this protein still resided in the nucleus (Fig. 7A). Nevertheless, we found that in cells containing HCV, as determined by fluorescence in situ hybridization (FISH) of viral RNA (see below), subcellular localization of cytoplasmic HNRNPK was altered and a fraction of HA-HNRNPKΔNLS was relocalized to ring-like structures that were not observed in the absence of HCV (Fig. 7A). While this ring-like pattern is indicative of LDs, in the light of our subcellular fractionation results (S8 Fig.) we assumed that this staining pattern likely corresponds to ER membranes surrounding LDs. Indeed, by determining the colocalization of LDs with the ER marker protein disulphide isomerase (PDI), HA-HNRNPKΔNLS and core were observed at LDs tightly surrounded by PDI-positive ER (Fig. 7B).

Since HNRNPK interacts with HCV RNA as determined by pull-down experiments, we determined subcellular localization of viral RNA by using fluorescence in situ hybridization (FISH) and colocalization of HCV RNA with HNRNPK as well as core. As shown in Fig. 7C, positive and negative strand HCV RNA as well as HNRNPK could be detected with high sensitivity. Specificity of HCV RNA detection of either polarity was corroborated by the absence of signal in cells that had been transfected with a replication-incompetent viral genome and analysis three days after transfection (S9 Fig.). By using this method we observed colocalization of core, HA-HNRNPKΔNLS and viral RNA of positive and negative polarity in ring-like structures, corresponding most likely to sites in close proximity of LDs where HCV assembly takes place (Fig. 7C and S10 Fig., respectively). Moreover, this colocalization was not found in case of HA-HNRNPK variants ΔKH1, ΔKH2 and ΔKI unable to suppress HCV assembly (S11 Fig.). Although cytoplasmic abundance of these variants was lower as compared to HA-HNRNPKΔNLS these results suggest that HNRNPK proteins capable to suppress HCV assembly are relocalized to putative HCV assembly sites. This localization is consistent with a role of HNRNPK in regulating virus particle production. In summary, we conclude that HNRNPK is a host cell factor determining efficiency of HCV particle production.

## Discussion

Several high-content screens have reported putative HDFs and HRFs promoting or restricting the HCV life cycle. Given the poor overlap of these screens, in the present study we combined a high-content RNA interference-based screen with an extensive meta-analysis, finally leading to the identification of 56 host cell factors affecting either early (entry and replication) or late steps (assembly and release) of the HCV life cycle. Bioinformatic analysis revealed significant enrichment of hits in distinct host cell pathways. These include the COPI system required for retrograde vesicular transport as well as LD homeostasis [56], or the SRP-dependent protein targeting machinery required for polyprotein processing (reviewed in reference [45]). Enrichments were also found for the epidermal growth factor receptor signaling pathway that plays a role for HCV entry (reviewed in reference [57]), and for LDL as well as plasma lipoprotein particle clearance pathways that are important for HCV assembly and release (reviewed in [3]). This over-representation of distinct cellular processes in the interaction network emphasizes their importance for the HCV life cycle.

We focused our analysis on HNRNPK that we identified as HRF limiting production of infectious HCV particles. In extension of earlier reports [48], [58], we demonstrate HNRNPK interaction with HCV RNA in the context of fully functional genomes. This interaction might be due to direct binding of HNRNPK to viral RNA or more indirectly, via interaction with the RNA binding proteins core or NS3. In support of the latter assumption we found that HNRNPK mutants impaired in interaction with HCV RNA also were impaired in interaction with these two viral proteins. In any case, the finding that HNRNPK mutants unable to pull down HCV RNA also have lost their capability to suppress virus production provides strong evidence for a direct mechanistic link. The relocalization of HNRNPK in HCV-infected cells to the vicinity of LDs, together with core and viral RNA, is in line with this proposed role in the HCV life cycle.

One plausible model explaining the underlying mechanism is that HNRNPK, via (direct or indirect) binding to HCV RNA, might regulate RNA availability for packaging into virions. In this model, HNRNPK would bind to viral genomes to feed them into a new cycle of RNA translation/replication, thus restricting viral RNA from incorporation into virus particles. Consequently, silencing of HNRNPK expression would increase the amount of viral RNA genomes available for packaging into nucleocapsids. In return, less genomes should enter a new cycle of RNA translation/replication. Indeed, by using cell lines containing a stable genotype 1 HCV replicon, previous studies reported a reduction of viral RNA replication upon knock-down of HNRNPK [59], [60], which is in line with this model. Although we also identified HNRNPK as a HDF in our entry/replication screen, in subsequent validation experiments, the impact of knock-down on HCV RNA replication was not statistically significant. Instead, we found that HNRNPK also plays a role in HCV entry. Moreover, HNRNPK has not been detected in affinity purified replication complexes [61] (D. Paul and R.B., unpublished), supporting the assumption that HNRNPK is not directly involved in HCV RNA replication. At first glance, this conclusion appears to contradict our model. However, we note that only a minor fraction of intracellular HCV RNA was coprecipitated with HNRNPK, suggesting that only a small proportion might be involved in virus assembly, which is consistent with the overall low assembly efficiency of HCV. Thus, releasing a HNRNPK-mediated block of assembly would have only a minor effect on the large pool of HCV RNA used for translation or replication and therefore, little effect on viral protein synthesis and RNA amplification, but well measurable effects on virus production.

Knock-down of HNRNPK primarily increased amounts of infectious HCV particles in culture supernatants as deduced from elevated levels of core protein and infectivity. The fact that intracellular amounts of core protein and infectivity were not affected argues that HNRNPK is not directly involved in virus release. Instead, we assume that HNRNPK determines assembly efficiency of infectious HCV particles. In that case, one might wonder why enhanced assembly did not lead to elevated amounts of infectious intracellular HCV particles. It has been reported that only a subfraction of intracellular particles are released whereas the rest is targeted for degradation [62]. Moreover, the same study showed that infectious HCV particles are rapidly released. Thus, accelerated assembly does not necessarily result in elevated amounts of intracellular infectious virus particles or core protein, consistent with our finding. Accelerated, but otherwise normal, assembly would also explain why neither specific infectivity of HCV particles, nor their biophysical properties as determined by rate zonal centrifugation were affected by HNRNPK silencing (M.P. and R.B. unpublished).

Apart from HCV, HNRNPK plays an important role in the life cycle of many other viruses where it acts either as dependency or restriction factor. For instance, HNRNPK was reported to stimulate transcription of the hepatitis B virus surface gene by binding to the respective enhancer in the viral genome [29]. This stimulation is abolished by APOBEC3 (apolipoprotein B mRNA editing enzyme catalytic polypeptide 3) that binds to and sequesters HNRNPK. In case of the human immunodeficiency virus, HNRNPK interacts with the accessory protein nef and activates signaling pathways that ultimately enhance transcription of the provirus [28]. Moreover, HNRNPK was reported to bind to a distinct stem-loop structure of the HIV-1 RNA genome, negatively affecting splicing [63]. In case of Sindbis virus, HNRNPK interacts with the subgenomic RNA [26], whereas in Enterovirus 71, it binds to a stem-loop structure in the 5′UTR [64]. Similar to what we observed for HCV, in these viruses the KI region as well as the RNA and DNA binding domains of HNRNPK are considered to be crucial for interaction with the viral RNAs. However, in all of these cases HNRNPK affects (directly of indirectly) viral replication whereas in the present study we describe a novel role of HNRNPK, i.e. regulating virus assembly. Although the detailed mechanistic aspects remain to be clarified, these examples illustrate that HNRNPK is usurped by evolutionary very distinct virus classes for various steps in their life cycles and presumably via different mechanisms. Our finding that HNRNPK is utilized by HCV to regulate specifically virus production adds a new facet to the complex regulation of virus - host cell interactions.

## Materials and Methods

### Cell culture and cell lines

For infection experiments the highly permissive cell lines Huh7.5 [65], Huh7-Lunet/CD81 [51], Huh7hp [40] or Huh7.5 FLuc were used, all derived from naïve Huh-7 cells [66]. Huh7.5 FLuc are derived from Huh7.5 cells by lentiviral transduction of the gene encoding for the *Firefly* luciferase (FLuc) by using a transduction approach described elsewhere [51] and culturing in the presence of 900 µg/ml G-418. Huh7.5 cells stably expressing wild type HA-tagged or untagged HNRNPK (Huh7.5 wt-HNRNPK, Huh7.5 HA-HNRNPK) or the mutant forms of HNRNPK (Huh7.5 HNRNPKΔNLS, Huh7.5 HNRNPKΔKNS, Huh7.5 HNRNPKΔctK, Huh7.5 HNRNPKΔKI, Huh7.5 HNRNPKΔKH1, Huh7.5 HNRNPKΔKH2 and Huh7.5 HNRNPKΔKH3) were generated by lentiviral transduction of the gene encoding for the HNRNPK variants by using the same transduction approach and culturing in the presence of 2 µg/ml puromycin. Cells were grown in Dulbecco's modified minimal essential medium (DMEM; Life Technologies, Frankfurt, Germany) supplemented with 2 mM L-glutamine, non-essential amino acids, 100 U/ml penicillin, 100 µg/ml streptomycin, and 10% fetal calf serum (complete DMEM).

### Viruses

The *Renilla* luciferase reporter virus (JcR2a; derived from plasmid pFK_I389_Core-3′-Jc1) encodes a *Renilla* luciferase that is fused N-terminally with the 16 N-terminal amino acid residues of the core protein and C-terminally with the foot-and-mouth disease virus (FMDV) 2A peptide [9]. Jc1 [30] and JcR2a viruses were produced by transient transfection of Huh7.5 cells with *in vitro* transcribed RNA as described previously [67]. The DENV genome used in this study contains a *Renilla* luciferase reporter gene and has been described elsewhere [68].

### *In vitro* transcription, electroporation of HCV RNAs and virus production

*In vitro* transcripts were generated by using 10 µg plasmid DNA that had been linearized by 1h-digestion with MluI. DNA was extracted with phenol and chloroform and, after precipitation with isopropanol, dissolved in RNase-free water. *In vitro* transcription reaction mixtures (total volume 100 µl) contained 80 mM HEPES (pH 7.5), 12 mM MgCl_2_, 2 mM spermidine, 40 mM dithiothreitol (DTT), 3.125 mM of each nucleoside triphosphate, 1 U/µl RNasin (Promega, Mannheim, Germany), 0.1 µg/µl of plasmid DNA, and 0.6 U/µl T7 RNA polymerase (Promega, Mannheim, Germany). After 2 h incubation at 37°C, 0.3 U/µl reaction mixture of T7 RNA polymerase was added and the reaction mixture was incubated for 2 h at 37°C. Transcription was terminated by adding 1.2 U of RNase-free DNase (Promega, Mannheim, Germany) per µg plasmid DNA and 30 min incubation at 37°C. RNA was extracted with acidic phenol and chloroform, precipitated with isopropanol at room temperature and dissolved in RNase-free water.

For virus production single-cell suspensions of Huh7.5 cells were prepared by trypsinization, washing with phosphate-buffered saline (PBS) and resuspension at a concentration of 1.5×10^7^ cells/ml in Cytomix [67] supplemented with 2 mM ATP and 5 mM glutathione. Ten µg *in vitro* transcripts were mixed with 400 µl cell suspension and transfected by electroporation using a GenePulser system (Bio-Rad, Hercules, CA) and cuvettes with a gap width of 0.4 cm (Bio-Rad) at 975 µF and 270 V. Cells were immediately diluted in complete DMEM and seeded. Virus-containing supernatants were collected, titrated and used for infection experiments.

### High-throughput siRNA screen

The siRNA library used for the primary siRNA screen (Ambion Silencer Extended druggable genome library V3) contains a total of 27,306 siRNAs targeting a subset of 9,102 human genes (listed in S2a and b Table), with three independent siRNAs per gene. A custom-made siRNA library from Dharmacon was used for the validation screen, with four independent siRNAs per gene (listed in S2c and d Table). The protocols for the high-throughput siRNA screening approach as well as statistical and bioinformatics analyses are described in Supplemental Experimental Procedures.

### Luciferase assay

Cells were lysed in luciferase lysis buffer (1% (v/v) Triton X-100, 10% (v/v) glycerol, 25 mM glycylglycine (pH 7.8), 15 mM MgSO_4_, 4 mM EGTA and 1 mM dithiothreitol). For dual luciferase measurement of *Firefly* and *Renilla* luciferase in 384- or 96-well microplates, cells were washed once with PBS, lysed directly on the plate in 20 µl (384-well plates) or 30 µl (96-well plates) luciferase lysis buffer per well and frozen at −80°C. Shortly before measurement lysates were allowed to thaw at RT for 30 to 60 min. Luciferase assay buffer (25 mM glycylglycin (pH 7.8), 15 mM K_2_PO_4_, (pH 7.8), 15 mM MgSO_4_, 4 mM EGTA, 1 mM DTT and 2 mM ATP), supplemented with 70 µM D-luciferin (P.J.K., Kleinblittersdorf, Germany), was added to each well using a Multidrop 384 dispenser (Thermo-Fisher, Martinsried, Germany), and plates were incubated for 5 min at RT in the dark. *Firefly* luciferase activity was measured for 0.1 sec in a Mithras LB940 multimode microplate reader (Berthold Technologies, Bad Wildbad, Germany). After addition of luciferase assay buffer, supplemented with 7.14 µM coelenterazine (P.J.K.), *Renilla* luciferase activity was measured for 0.5 sec using a 475 nm filter in the same multiwell reader.

### Electroporation of siRNAs

Replication assays were performed with Huh7-Lunet cells, infection assays with Huh7.5 or Huh7.5Fluc cells. Single-cell suspensions were prepared by trypsinization, washed with phosphate-buffered saline (PBS) and resuspended at a concentration of 10^7^ Huh7-Lunet cells or 1.5×10^7^ Huh7.5 cells per ml in Cytomix [69] supplemented with 2 mM ATP and 5 mM glutathione. For knock-down experiments, 100 µl cell suspension was mixed with 2.5 µM siRNA and transfected by electroporation using a GenePulser system (Bio-Rad, Hercules, CA) and a 0.2 cm gap cuvette (Bio-Rad, Hercules, CA) at 500 µF and 166 V. Cells were immediately diluted in complete DMEM and seeded as required for the given assay. SiRNAs are listed in Supplemental Experimental Procedures.

### siRNAs

The used siRNA (Eurofins MWG Opern, Ebersberg, Germany) had the following sequences (only sequences of the sense strands are given): siHNRNPK#1 5′-GCAAGAAUAUUAAGGCUCU-3′; siHNRNPK#2 5′-GGUCGUGGCUCAUAUGGUG-3′; siHNRNPK#3 5′-UGACAGAGUUGUUCUUAUU-3′; siHNRNPK#4 5′-UAAACGCCCUGCAGAAGAU-3′; siHNRNPK3′NTR 5′-CGUUAUUGUUGGUGGUUUA-3′; siHNRNPKcontr. 5′-GAAAGUUUUUCUAAGACUA-3′; siRluc 5′-GUAGCGCGGUGUAUUAUAC-3′; siApoE 5′-CUAGUUUAAUAAAGAUUCA-3′; siContr. 5′-UGGUUUACAUGUCGACUAA-3′.

### Western blot analysis

Cells in a confluent 24-well cell culture plate were washed once with PBS and harvested in 100 µl per well of 2 x protein sample buffer (200 mM Tris, pH 8.8, 5 mM EDTA, 0.1% Bromophenolblue, 10% sucrose, 3% SDS, 2% β-mercaptoethanol) followed by an incubation at 37°C for 30 min with 50–75 U benzonase (Merck, Darmstadt, Germany) and heated for 5 min at 95°C. Proteins were separated by SDS-polyacrylamide gel electrophoresis and electrotransferred onto polyvinylidene membranes (Perkin Elmer; Rodgau, MA), followed by blocking with PBS-0.5%Tween 20 containing 5% dried milk for 2 h prior to 1h-incubation with specific antibodies, each diluted 1∶1,000 in PBS containing 1% dry milk. Membranes were washed 3 times with PBS supplemented with 0.5% Tween 20 and incubated for 1 h with either horseradish-peroxidase-conjugated rabbit- or mouse-specific secondary antibodies (Sigma-Aldrich, Steinheim, Germany) diluted 1∶10,000. Membranes were developed by using the Western Lightning Plus-ECL reagent (Perkin Elmer; Rodgau, MA) and bands were visualized on Amersham Hyperfilm ECL (GE Healthcare Life Sciences, Uppsala, Sweden).

### Production of lentiviral vectors and stable cell lines

For production of the lentiviral vectors, 2.4×10^6^ 293T cells were seeded per 6 cm-diameter dish in a volume of 4 ml DMEM complete one day prior to transfection by using the JetPEI transfection kit (Polyplus Transfection, NY, USA) as recommended by the manufacturer. Production of the lentiviral vectors has been described elsewhere [70]. In brief, 5 µg of the respective pWPI plasmid, 5 µg of the packaging plasmid (pCMV-R8.74) and a VSV envelope glycoprotein expression plasmid (pMD.G) were transfected into 293T cells. After 24 h transfection medium was replaced by 4 ml fresh DMEM complete and after an additional 24h-incubation, lentivirus particles-containing supernatant was harvested. Supernatant was filtered through a 0.45 µm filter and 1.5 ml of the filtrate was used to infect Huh7.5 target cells that had been seeded at a density of 2×10^5^ cells/well of a 6-well plate 24 h before inoculation. Transduction of target cells with the lentiviral particles was performed in total three times to achieve high number of integrates and thus high expression levels. Transduced cells were subjected to selection by using medium containing the appropriate drug.

### Antibodies

Mouse monoclonal antibody recognizing NS3 of the JFH-1 isolate (NS3-2E3) was generated in co-operation with H. Tang, Florida State University, USA. The mouse monoclonal antibody 9E10 recognizing NS5A domain III of the HCV isolates Con1 and JFH-1 was a kind gift of C. M. Rice (Rockefeller University, New York, USA). The mouse monoclonal antibody recognizing HCV core protein (C7/50) was kindly provided by D. Moradpour (University of Lausanne, Switzerland). The rabbit polyclonal antibody recognizing NS2 (NS2-1519) has been described earlier [71]. Mouse monoclonal antibody against *Renilla* Luciferase was obtained from Chemicon Millipore (USA). Mouse monoclonal antibodies against Lamin and GAPDH were obtained from Santa Cruz Biotechnology (Heidelberg, Germany). The polyclonal rabbit antibody recognizing HNRNPK was purchased from Acris Antibodies (Herford, Germany). Primary antibodies against the HA-tag (mouse, H3663), HA-specific agarose beads (A2095), rabbit polyclonal antibody reacting with protein disulfide isomerase (PDI) as well as secondary horse radish peroxidase-conjugated antibodies were purchased from Sigma-Aldrich. Chicken polycloclonal antibody against the HA-tag was obtained from Abcam (Cambridge, UK)

### Immunoprecipitation

For immunoprecipitation 4×10^6^ Huh7.5 cells were electroporated and seeded into a 10 cm-diameter dish. After 48 h, cells were washed with ice-cold PBS and lysed for 1 h on ice in ice-cold lysis buffer (20 mM Tris-HCl, pH 7.5, 0.5% Nonidet P-40, 1 mM sodium deoxycholate, 10 mM NaF, 2 mM EDTA, protease inhibitor cocktail (Roche, Mannheim, Germany), 0.5 mM DTT, 2.5 U/ml RNasin), supplemented with either 150 mM NaCl for co-immunoprecipitation of RNA or 100 mM NaCl for co-immunoprecipitation of viral proteins. Lysates were cleared by centrifugation at 13,000 x g for 30 min at 4°C. Non-specifically binding proteins contained in the supernatants were removed by 1 h-incubation with prot-A agarose at 4°C, followed by centrifugation at 6,000×g for 5 min. Supernatants were used for immunoprecipitation (3 h at 4°C) by using mouse monoclonal anti-HA-coated agarose beads (Sigma-Aldrich) followed by washing 4 times with ice-cold lysis buffer. HA-peptide (Sigma-Aldrich) was used to elute bound antigen. One half of the immunocomplex was used for RNA isolation by using the Nucleo Spin RNAII Kit (Macherey-Nagel, Düren, Germany) as recommended by the manufacturer. The other half of the lysates was dissolved in 60 µl 4 x protein sample buffer (400 mM Tris-HCl pH 8.8, 10 mM EDTA, 0.2% bromophenolblue, 20% sucrose, 3% SDS and 2% β-mercaptoethanol). Immuno-captured proteins were separated by electrophoresis into 10% polyacrylamide gels and dried gels were subjected to autoradiography using BioMax MS films (Kodak, Rochester, MN). For quantification, gels were either analyzed by phosphoimaging or different exposures of the films were scanned and subjected to densitometry by using the QuantityOne software (Bio-Rad, Munich, Germany).

### Quantification of RNA by RT-qPCR

RT-qPCR reactions were carried out using the two-step real-time RT-PCR approach. In a first step RNA was reverse transcribed into cDNA using the Multiscribe reverse transcriptase (Applied Biosystems) according to the manufacturer's protocol. Synthesized cDNAs were directly used for real-time PCR or stored at −80°C until further use. The final volume of the real-time PCR reaction mix was 15 µl and contained the following components: 7.5 µl 2x Green DYE master mix (P.J.K., Kleinbittersdorf, Germany), 1.5 µl primer mix (5 µM each), 3 µl ddH_2_O and 3 µl cDNA. Reactions were performed on an ABI PRISM 7000 Sequence Detection System using the following settings: 95°C: 10 min → 40x [95°C: 30 sec → 58°C: 60 sec → 72°C: 60 sec]. For each primer set reactions were carried out in triplicates using HNRNPK-specific primers (forward: TTCAGTCCCAGACAGCAGTG; reverse: TCCACAGCATCAGATTCGAG). The ΔΔCT method [72] was used to calculate the relative expression levels. GAPDH forward: GAAGGTGAAGGTCGGAGTC, reverse: GAAGATGGTGATGGGATTTC.

### Indirect immunofluorescence

Cells were washed once with PBS and fixed with 500 µl of 4% paraformaldehyde for 20 min at RT followed by three times washing with PBS. Fixed cells were permeabilized by 5 min incubation in 500 µl of 0.5% Triton X-100 in PBS and washed 3 times with PBS. Immunostaining was performed by using the rabbit polyclonal HNRNPK antibody (1∶200 dilution), mouse monoclonal *Renilla* luciferase antibody (1∶1,000 dilution), mouse monoclonal core antibody (1∶200 dilution) or rabbit polyclonal PDI antibody (1∶200 dilution). Antibodies were diluted in PBS supplemented with 3% bovine serum albumin (BSA). After 60 min incubation and three times washing with PBS, cells were incubated with secondary antibodies conjugated to Alexa-Fluor 546, 488 or 405 at a dilution of 1∶1,000 in PBS containing 3% BSA for 60 min in the dark. Nuclei were stained with DAPI (Sigma-Aldrich). LDs were stained with HCS LipidTOX Deep Red neutral lipid stain (Molecular Probes). Coverslips were mounted on glass slides with Fluoromount G (Southern Biotechnology Associates, Birmingham, USA) and samples examined using a Leica SP2 confocal laser scanning microscope (Leica, Wetzlar, Germany) or Perkin Elmer spinning disk confocal microscope. Images were edited and merged by using the ImageJ software package (Rasband, W.S., ImageJ, U. S. National Institutes of Health, Bethesda, Maryland, USA; http://rsb.info.nih.gov/ij/). For Fig. 7B, images were deconvolved with the AutoQuantX3 using blind deconvolusion. Reconstructed 3D images were created using the Imaris 7.7.2 software.

RNA in situ hybridization and indirect immunofluorescence.

Cells were washed once with PBS and fixed with 500 µl of 4% paraformaldehyde for 20 min at RT, followed by three times washing with PBS. Fixed cells were permeabilized by 1 h incubation in 500 µl 70% ethanol and washed 3 times with PBS. Positive and negative-strand HCV RNA was detected by FISH using the QuantiGene ViewRNA ISH Cell Assay (Affymetrix) as recommended by the manufacturer. Immunostaining was performed by using a chicken polyclonal antibody recognizing the HA-tag (1∶200 dilution) and a mouse monoclonal antibody recognizing HCV core protein (C7/50) (1∶200 dilution) after blocking the cells with 20% goat serum in PBS/0.01% Triton X-100. Antibodies were diluted in PBS, supplemented with 10% goat serum. After 60 min incubation cells were washed three times with PBS and incubated with secondary antibodies conjugated to Alexa-Fluor 546 or 488 at a dilution of 1∶1,000 in PBS containing 10% goat serum for 60 min in the dark. Nuclei were stained with DAPI (Sigma-Aldrich). Coverslips were mounted on glass slides with Fluoromount G (Southern Biotechnology Associates, Birmingham, USA) and samples examined using a Leica SP2 confocal laser scanning microscope (Leica, Wetzlar, Germany). Images were edited and merged by using the ImageJ software package (Rasband, W.S., ImageJ, U. S. National Institutes of Health, Bethesda, Maryland, USA; http://rsb.info.nih.gov/ij/).

### Cell proliferation assay

The cell proliferation reagent WST-1 (Roche) was used as recommended by the manufacturer.

### Lipid droplet enrichment

LDs were enriched as described earlier [73]. In brief, Huh7.5 cells were electroporated with 10 µg full length in vitro transcripts of the JFH-1 isolate and treated with 200 µM oleic acid (Sigma-Aldrich) for 16 h prior to harvesting. Cells were harvested 72 h post electroporation by scraping into PBS containing 5 mM EDTA. Cells were pelleted by centrifugation, resuspended in 1.3 ml HLM buffer (20 mM Tris, pH 7.4, 1 mM EDTA, protease inhibitor cocktail) and incubated on ice for 10 min. Cells were disrupted by douncing and nuclei were removed by centrifugation at 1,000×g for 10 min at 4°C. The post nuclear fraction was mixed with 0.5 volumes 60% (wt/vol) sucrose dissolved in HLM buffer and overlaid with 5 ml of HLM-5% (wt/vol) sucrose, followed by 5 ml of HLM buffer. The gradient was centrifuged at 13,000×g for 30 min at 4°C and then the centrifuge was stopped without brake. Four hundred µl of the LD fraction was collected from the top of the gradient and proteins were precipitated by using an acidified aceton/methanol mixture. Pellets were resuspended in SDS-containing 2x protein sample buffer and analyzed by Western-blot using a 10% polyacrylamide gel.

### Determination of virus titers, HCV core protein and ApoE amounts

Huh7.5 cells were co-electroporated with 2.5 µM of siRNA and 5 µg of Jc1 RNA and seeded into 6-well plates. To determine the amounts of extracellular infectivity, supernatant was harvested 72 h after electroporation, filtered through a 0.45 µm-pore-size filter and stored at 4°C. To quantify amounts of intracellular infectivity, cells were rinsed three times with PBS and scraped into 0.5 ml PBS. Cells were pelleted by centrifugation for 5 min at 700×g, resuspended in 0.5 ml of complete DMEM and subjected to three freeze-thaw cycles. Cell debris was removed by centrifugation for 10 min at 20,000×g. Virus titers were determined by limiting-dilution assay using Huh7.5 target cells and staining of the NS3 protein with the 2E3 antibody as described elsewhere [70]. To determine amounts of core protein, cells and supernatant were subjected to three freeze-thaw cycles and diluted to a final concentration of 0,5% Triton X-100/PBS/protease inhibitor (Roche). Lysates were cleared by centrifugation at 20,000×g for 10 min at 4°C. HCV core protein was quantified using a commercial chemiluminescent microparticle immunoassay (CMIA) (6L47, ARCHITECT HCV Ag Reagent Kit, Abbott Diagnostics, Abbott Park, USA) according to the instructions of the manufacturer. To determine ApoE amounts, cells and supernatant were subjected to three freeze-thaw cycles. ApoE protein was quantified using a commercial Human Apo E ELISA Kit (Cell Biolabs, USA) according to the instructions of the manufacturer.

### Preparation of retroviral pseudoparticles and flow cytrometry

HIV-based pseudotypes containing HCV envelope glycoproteins of the Con1 isolate were generated by transfection of 293T cells. Briefly, 6×10^7^ cells were seeded into 10 cm-diameter dishes in a volume of 8 ml DMEM complete (Life Technologies) one day prior to transfection by using the JetPEI transfection kit (Polyplus Transfection, NY, USA) as recommended by the manufacturer. Production of the lentiviral vectors has been described elsewhere [74]. In brief, 5 µg of the respective HCV envelope protein expression construct (pcDNAΔC-E1E2), the HIV Gag-Polymerase construct (pHIT60) and the VENUS-transducing lentiviral vector were transfected into 293T cells. After 24 h transfection medium was replaced by 8 ml fresh DMEM complete and after an additional 24 h and 48h-incubation, HCVpp-containing supernatant was harvested. Supernatant was filtered through a 0.45 µm filter and overlaid with 4 ml 20% (wt/vol) sucrose, followed by centrifugation at 26,000×g for 2 h at 4°C. Pellets were suspended in DMEM complete and used for infection of Huh7.5 cells that had been seeded into 6-well plates for 72 h. Cells were detached by adding trypsin, fixed with 500 µl of 2% paraformaldehyde for 1 h at RT followed by washing with PBS. Samples were analyzed by flow cytometry and data were processed by using the FlowJo Analysis Software package (Tree Star, USA).

## Supporting Information

S1 Fig **Results of the primary siRNA screen.** (**A**) siRNA results of the primary screen for entry/replication (left) and assembly/release (right). The screen was performed in three replicates; all replicates were used to compute z-scores for each siRNA (black dots). Hit siRNAs were defined by a z-score ≤−2 (green line) for host dependency factors (HDFs; green dots) or z-score ≥ 2 (red line) for host restriction factors (HRFs; red dots) and only when this was the case for at least two siRNAs per gene. We identified 78 genes as HDFs and 29 genes as HRFs of the HCV lifecycle. Biological processes (**B**) and molecular functions (**C**) annotated to all hits of the primary screen. The diagrams show all UniProtKB keywords that are annotated to at least two hits, allowing multiple keywords per hit. Data were obtained using the DAVID 6.7 software package (see Experimental Procedures and S1 Table).
(TIF)

S2 Fig **Network representation of host factors that are of relevance for the HCV life cycle.** The network displays host cell factors identified in our study (rectangular nodes) and in previous studies (oval or rectangular nodes with black borders). Oval nodes depict host factors that were either not investigated or not confirmed in our study. Confirmed HDFs are specified in green boxes, HRFs in red boxes and factors with unclear function (HDF and/or HRF) in yellow boxes. Lines connecting two nodes indicate proteins with known physical interaction. Selected biological processes for which we found a significant enrichment of HCV host factors are highlighted by coloured areas and listed with their Gene Ontology identifiers below the network in the correspondingly coloured box (see also Supplemental Experimental Procedures).
(TIF)

S3 Fig **Impact of HCV on HNRNPK transcription and protein abundance.** (**A**) Amount of HNRNPK mRNA is not affected by HCV infection. Huh7.5 cells were infected with Jc1 (MOI  = 10 TCID_50_/ml), harvested at given time points post infection and total RNA was isolated. Amounts of HNRNPK mRNA and viral RNA were analyzed by RT-qPCR. GAPDH mRNA was used for normalization. HNRNPK mRNA levels in infected cells were compared to those detected in non-infected Huh7.5 cells that were set to 1. Bars represent the mean ±SD of two independent experiments. (**B**) Amount of HNRNPK protein is not altered during HCV infection. Huh7.5 cells were electroporated with 10 µg of genomic HCV RNA (isolate Jc1). Cells were harvested 72 h later and lysates were analyzed by Western blot using antibodies specified in the right. To verify HCV infection, lysates were probed for NS3 and NS2. GAPDH was used as loading control.
(TIF)

S4 Fig **Restriction of assembly/release by HNRNPK is HCV genotype independent.** Forty-eight hours post silencing, siRNA-transfected cells were infected with JcR2a (**A**) or H77R2a (**B**) or Con1R2a (**C**) reporter virus (MOI  = 0.4 TCID_50_/ml). To determine the impact of knock-down on viral entry/replication (grey bars), cells were lysed 48 h post infection. To measure knock-down impact on virus production (black bars), supernatants of transfected cells were used to inoculate Huh7.5 cells that were lysed 72 h later. Virus replication was quantified by measuring *Renilla* luciferase activity (relative light units, RLU) and values were normalized to cell viability. Non-targeting control siRNA (siContr.) was set to 100% (dotted line). SiRNAs targeting the Renilla luciferase sequence in the reporter virus genomes served as positive control. Bars represent the mean ±SD of at least two independent experiments. (**D**) HNRNPK knock-down also enhances production of JFH-1 wildtype virus. Huh7.5 cells were co-electroporated with JFH-1 RNA and either a mix of HNRNPK-specific siRNAs (#1–4) or a control siRNA. After 72 h titers of infectious virus contained in the supernatant (extracellular) or cell lysate (intracellular) were determined by TCID_50_ assay. Shown is the average of three independent experiments ±SD. Knock down efficiency was determined by Western blot; a representative blot is shown. In all panels, statistical analysis was performed by using Student's t-test, with reference to siContr. ***, P-value ≤0.0005; **, P-value ≤0.005; *, P-value ≤0.05; ns, non-significant.
(TIF)

S5 Fig **Mapping of HNRNPK domains required for restriction of HCV particle production.** (**A**) Schematic of siRNA targeting sites in HNRNPK deletion mutants. Target sites of siHNRNPK#1 to #4 are indicated with an asterisk. Blue and red color indicate present or absent target sites, respectively. (**B-F**) Characterization of HNRNPK mutants ΔcKBR, ΔKNS, ΔKI, ΔKH3 or ΔKH1 for their efficiency to restrict HCV particle production, respectively. Silencing was performed by using 2.5 µM of each indicated siRNA. Cells were infected with the HCV *Renilla* luciferase reporter virus JcR2a (MOI  = 0.4 TCID_50_/cell) after a 48 h-silencing period. To detect cellular genes involved in HCV entry/replication (grey bars), cells were lysed 48 h post infection and *Renilla* luciferase activity was measured. To evaluate the effect of silencing on assembly/release, virus-containing supernatant was used for infection of naïve Huh7.5 cells (black bars). Replication was determined by *Renilla* luciferase assay in lysates of cell prepared 72 h post inoculation. Values (relative light units, RLU) were normalized to cell viability and the non-targeting control siRNA (siContr.) that was set to 100% (R2 value, black dotted line). Bars represent the mean ±SD of four independent experiments. To quantify suppression of virus production by ectopic HNRNPK expression, mean values obtained with siRNA HNRNPK#1 to #4 (dotted horizontal red line; value R1) and siHNRNPK-3′NTR targeting only endogenous HNRNPK were compared with each other (value R3). Values were normalized to the ones obtained with the siContr. that was set 100% (value R2; dotted horizontal black line). Thus, R3 values reflect the relative suppression of HCV particle production achieved with ectopically expressed HNRNPK. ***, P-value ≤0.0005; **, P-value ≤0.005; *, P-value ≤0.05; ns, non-significant. Statistical analysis was performed by using Student's t-test, referred to the non-targeting control siRNA (siContr.).
(TIF)

S6 Fig **Interaction of HNRNPK with HCV core and NS3.** Huh7.5 cells stably expressing wild type or HA-tagged HNRNPK were electroporated with a subgenomic JFH1 replicon (HCVsg) or genomic HCV RNA (HCV). Cells were harvested 48 h post electroporation and lysates were used for coimmunoprecipitation using HA-specific antibody-coated agarose beads. Immunocomplexes and total cell lysates (corresponding to 10% and 1% of the total fraction, respectively) were analyzed by immunoblot (IB) using antibodies specified in the left. GAPDH was used as loading control. Endogenous HNRNPK is indicated with red asterisks. Numbers in the left refer to the positions of molecular weight protein standards.
(TIF)

S7 Fig **Interaction of HNRNPK deletion mutants with HCV core and NS3.** Huh7.5 cells stably expressing wild type, HA-tagged HNRNPK or HA-tagged HNRNPK deletion mutants (HNRNPKΔKH1, ΔKH2 and ΔNLS) were electroporated with 5 µg genomic HCV RNA. Cells were harvested 48 h later and lysates were used for coimmunoprecipitation using HA-specific antibody-coated agarose beads. Immunocomplexes and total cell lysates (corresponding to 10% and 1% of the total fraction, respectively) were analyzed by immunoblot (IB) using antibodies specified in the left. GAPDH was used as loading control. Numbers in the left refer to the positions of molecular weight protein standards.
(TIF)

S8 Fig **HNRNPK does not associate with LDs in HCV-containing cells.** Huh7.5 cells were electroporated with genomic JFH1 (HCV) RNA or mock-transfected. Cells were harvested 72 h later and lipid droplets (LD) were isolated from total cell lysate by differential centrifugation. Total cell lysate as well as LD-fractions were analyzed by Western blot using antibodies specified in the right. Purity of LD fractions was determined by using antibodies detecting core, calnexin, DDX3 and GAPDH.
(TIF)

S9 Fig **Specificity of HCV positive-stand and negative-strand RNA detection.** (**A, B**) Huh7hp cells were electroporated with either 5 µg subgenomic JHF1 RNA or a replication-deficient JFH1 mutant containing mutations at the NS5B polymerase active site (sgJFHdelGDD). Seventy two hours after transfection, cells were fixed and HCV RNA was detected by fluorescent in situ hybridization (FISH) using the QuantiGene ViewRNA ISH Cell Assay (Affymetrix). Nuclear DNA was stained with DAPI. Note that Huh7hp cells were used instead of Huh7.5 cells because of the lower background staining obtained by FISH. (**A**) Positive-strand HCV RNA detection; **B**) Negative-strand HCV RNA detection.
(TIF)

S10 Fig **Quality control of strand-specific detection of HCV RNA and colocalization of HNRNPK with core and viral RNA.** (**A**) Huh7hp cells stably expressing untagged HNRNPK were mock-electroporated and stained for the HA-tag, core as well as positive- and negative-strand HCV-RNA. Seventy two hours after transfection, cells were fixed and stained. Nuclear DNA was stained with DAPI. Note the absence of background staining. Note that Huh7hp cells were used instead of Huh7.5 cells because of the lower background staining obtained by FISH. (**B**) Huh7hp cells stably expressing HA-tagged HNRNPKΔNLS were electroporated with 5 µg genomic JFH1 (HCV) RNA and fixed 72 h later. Tagged HNRNPKΔNLS was detected by HA-specific immunofluorescence staining. HCV negative-strand RNA was detected by FISH using the QuantiGene ViewRNA ISH Cell Assay (Affymetrix). Enlargements of the sections shown in the bottom are indicated by white squares in the corresponding top panels. Images were acquired with a confocal microscope; scale bar refers to 10 µm.
(TIF)

S11 Fig **Subcellular distribution of HNRNPK deletion mutants in HCV-containing cells.** Huh7hp cells stably expressing the HA-tagged variants HNRNPKΔNLS, HNRNPKΔKH1, HNRNPKΔKH2 or HNRNPKΔKI were electroporated with genomic JFH1 RNA and fixed 48 h later. HA-tagged HNRNPK variants were detected by immunofluorescence staining using a HA-specific antibody and Core was detected by using a monoclonal antibody. Inserts in the lower left of the images show magnifications of boxed areas. Scale bars represent 10 µm or 5 µm in regular and magnified images, respectively.
(TIF)

S1 Table **Summary of the HCV druggable siRNA HCV screen (primary and validation screen), meta-analysis of the screen and bioinformatic analysis of identified hits.** This table contains 5 sheets, identified by a letter and a short name. **Sheet A**) Overall screen results: Summary of the primary and validation screen. This main table summarizes the results of the primary and validation screens and lists all confirmed factors. **Sheet B**) Meta-analysis: Summary of the identified additional candidates that were included in the validation screen. **Sheet C**) GO – biological process (BP) term enrichments (BP) of all validated hits. **Sheet D**) UniProt Keywords Primary Hits: UniProtKB SwissProt keywords from biological process (BP) and molecular function (MF) categories for all hits from the primary screen. These data are the basis for S1B Fig. and C. Sheet E) Host factor interactions: Binary protein-protein interactions between confirmed hits and previously published host factors. A more detailed description of the individual columns of each data sheet is provided in the overview sheet.
(XLS)

S2 Table **Results of the HCV primary and validation siRNA screen.** This table summarizes siRNAs used in our HCV siRNA screen, along with mean z-scores and p-values. For each tested siRNA, the gene ID and gene symbol, siRNA sequence, mean z-score, and p-value are listed. **Sheet A**) Entry and replication part of the primary siRNA screen (Ambion Silencer Extended druggable genome library V3). **Sheet B**) Assembly and release part of the primary siRNA screen (Ambion Silencer Extended druggable genome library V3). **Sheet C**) Entry and replication part of the validation siRNA screen (custom-made siRNA library from Dharmacon). **Sheet D**) Assembly and release part of the validation siRNA screen (custom-made siRNA library from Dharmacon).
(XLSX)

S1 Methods **Supplemental Materials and Methods.**
(DOC)
