# Supplementary figures and images for: Identification of HNRNPK as Regulator of Hepatitis C Virus Particle Production
Source: PLoS Pathog. 2015 Jan 8;11(1):e1004573. doi: 10.1371/journal.ppat.1004573 (PMC4287573; doi:10.1371/journal.ppat.1004573)

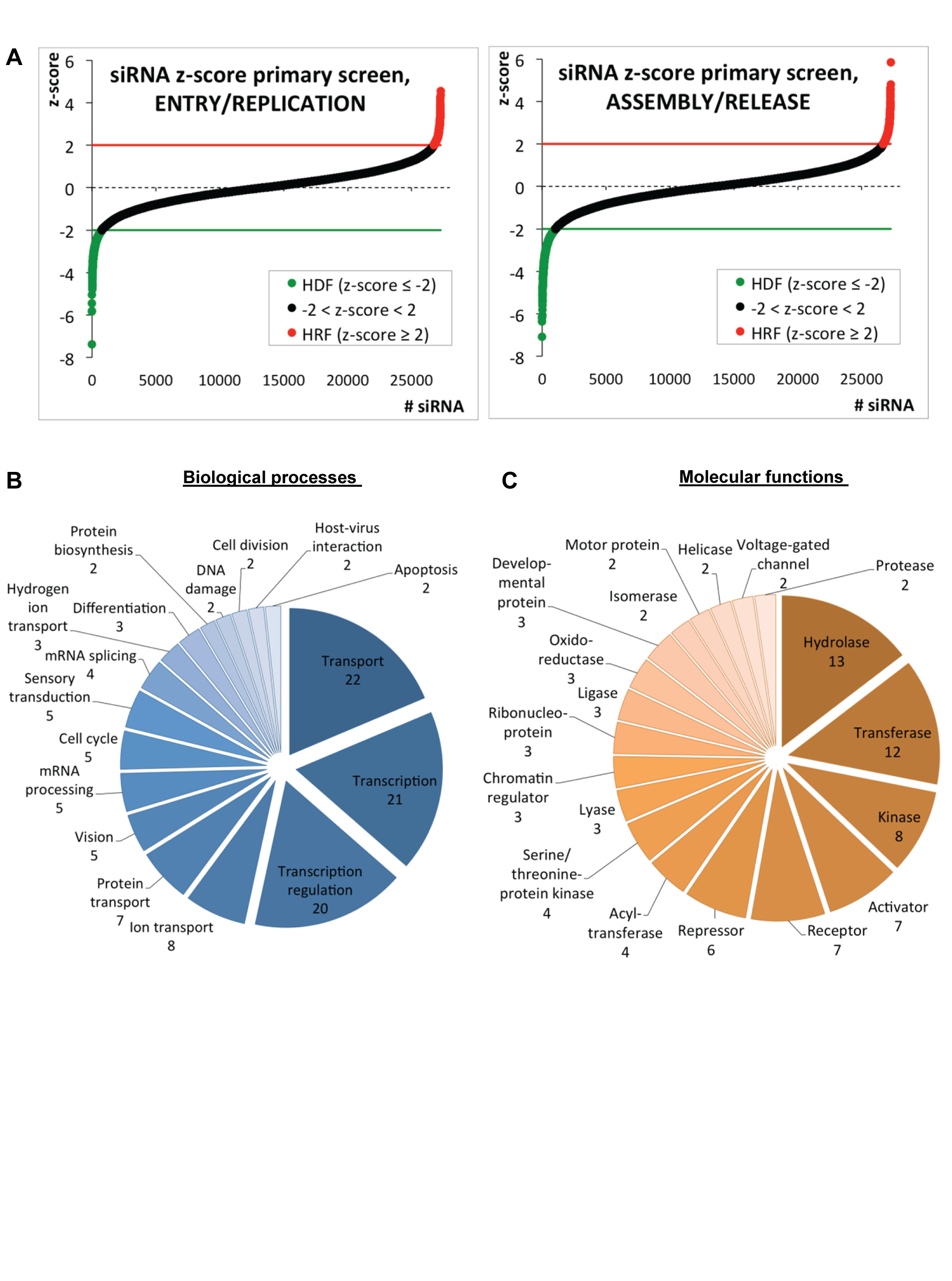

Supplement: S1 Fig — Results of the primary siRNA screen. (A) siRNA results of the primary screen for entry/replication (left) and assembly/release (right). The screen was performed in three replicates; all replicates were used to compute z-scores for each siRNA (black dots). Hit siRNAs were defined by a z-score ≤−2 (green line) for host dependency factors (HDFs; green dots) or z-score ≥ 2 (red line) for host restriction factors (HRFs; red dots) and only when this was the case for at least two siRNAs per gene. We identified 78 genes as HDFs and 29 genes as HRFs of the HCV lifecycle. Biological processes (B) and molecular functions (C) annotated to all hits of the primary screen. The diagrams show all UniProtKB keywords that are annotated to at least two hits, allowing multiple keywords per hit. Data were obtained using the DAVID 6.7 software package (see Experimental Procedures and S1 Table). (TIF) [file ppat.1004573.s001.tif]

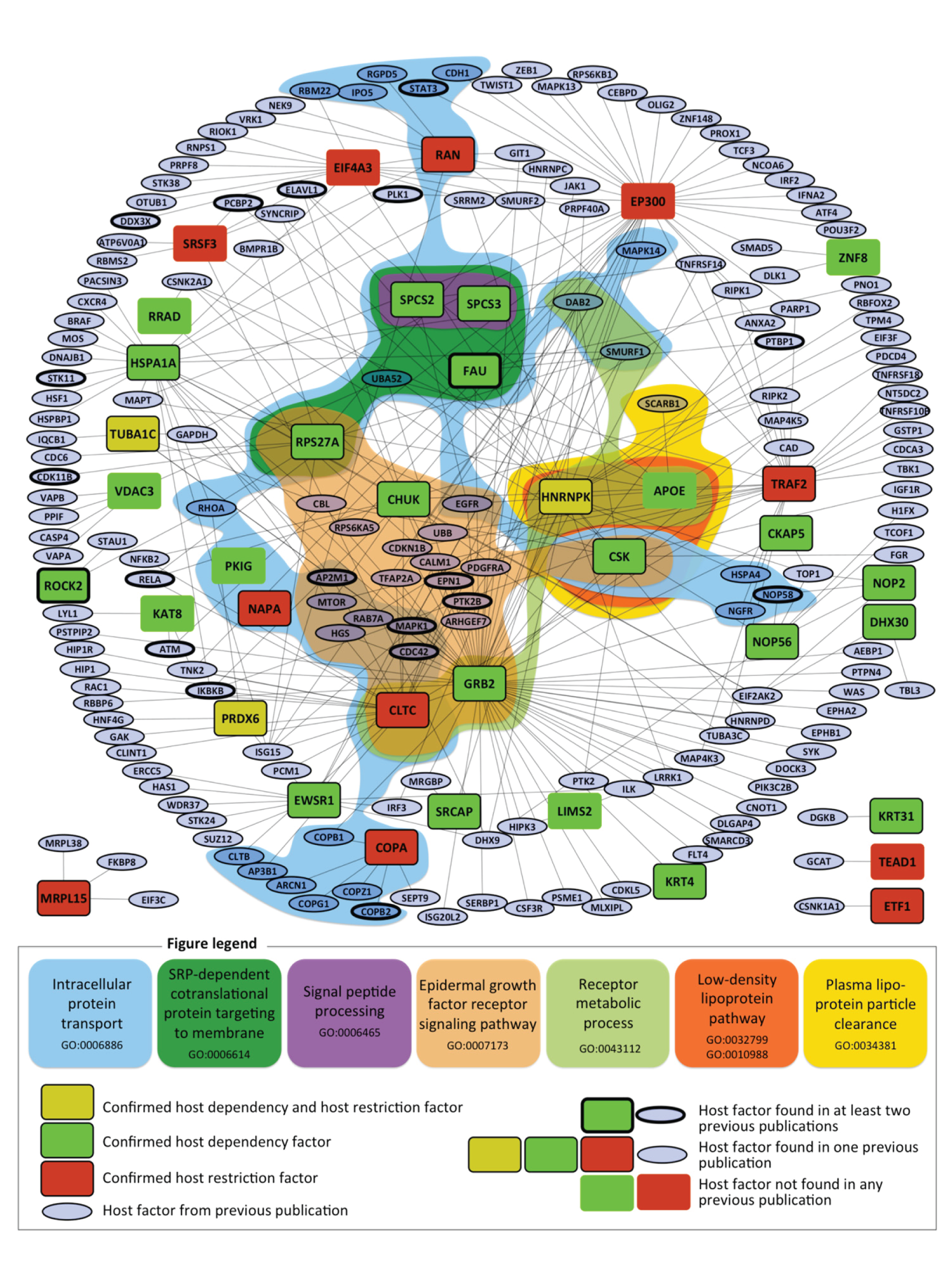

Supplement: S2 Fig — Network representation of host factors that are of relevance for the HCV life cycle. The network displays host cell factors identified in our study (rectangular nodes) and in previous studies (oval or rectangular nodes with black borders). Oval nodes depict host factors that were either not investigated or not confirmed in our study. Confirmed HDFs are specified in green boxes, HRFs in red boxes and factors with unclear function (HDF and/or HRF) in yellow boxes. Lines connecting two nodes indicate proteins with known physical interaction. Selected biological processes for which we found a significant enrichment of HCV host factors are highlighted by coloured areas and listed with their Gene Ontology identifiers below the network in the correspondingly coloured box (see also Supplemental Experimental Procedures). (TIF) [file ppat.1004573.s002.tif]

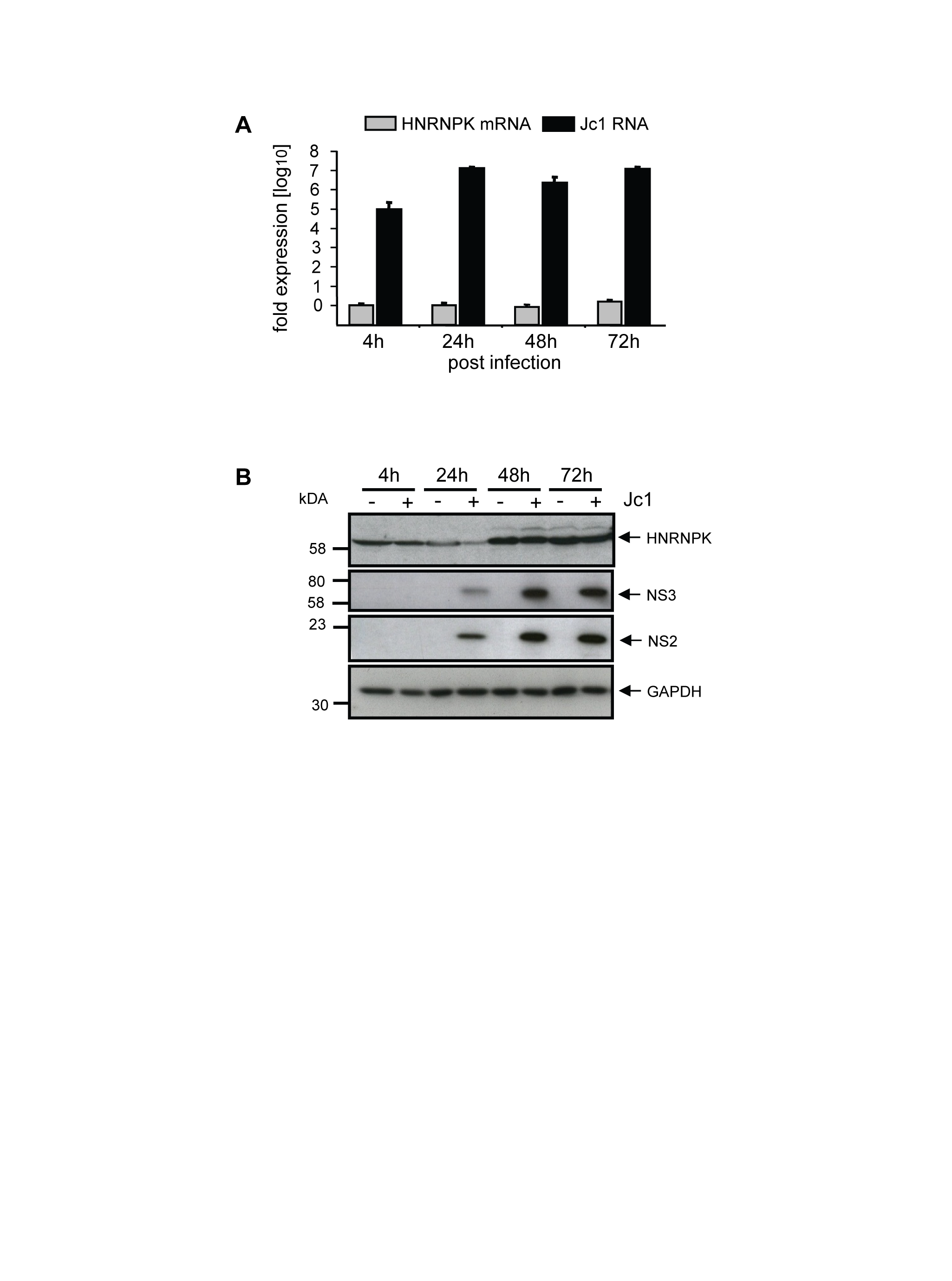

Supplement: S3 Fig — Impact of HCV on HNRNPK transcription and protein abundance. (A) Amount of HNRNPK mRNA is not affected by HCV infection. Huh7.5 cells were infected with Jc1 (MOI = 10 TCID50/ml), harvested at given time points post infection and total RNA was isolated. Amounts of HNRNPK mRNA and viral RNA were analyzed by RT-qPCR. GAPDH mRNA was used for normalization. HNRNPK mRNA levels in infected cells were compared to those detected in non-infected Huh7.5 cells that were set to 1. Bars represent the mean ±SD of two independent experiments. (B) Amount of HNRNPK protein is not altered during HCV infection. Huh7.5 cells were electroporated with 10 µg of genomic HCV RNA (isolate Jc1). Cells were harvested 72 h later and lysates were analyzed by Western blot using antibodies specified in the right. To verify HCV infection, lysates were probed for NS3 and NS2. GAPDH was used as loading control. (TIF) [file ppat.1004573.s003.tif]

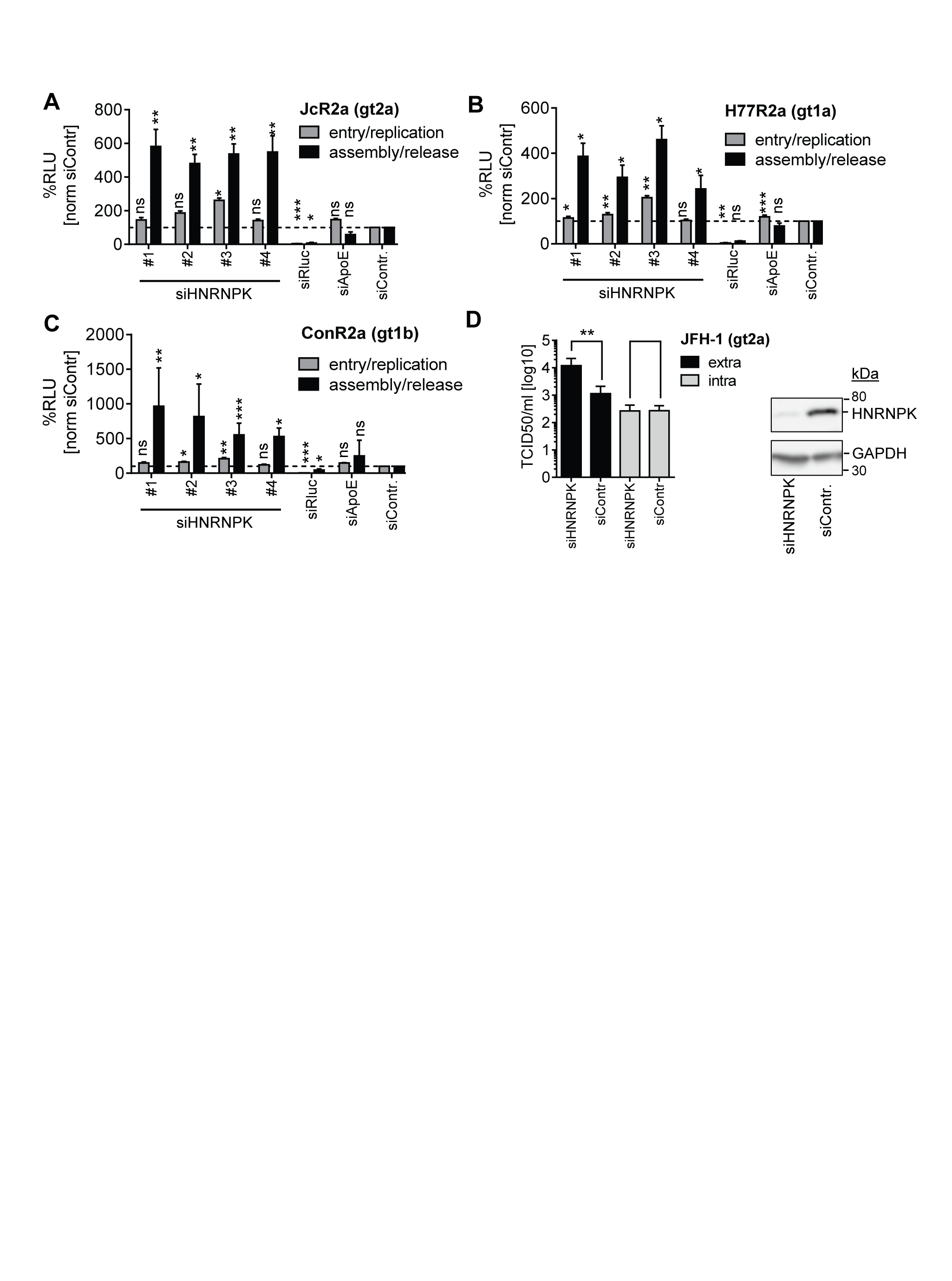

Supplement: S4 Fig — Restriction of assembly/release by HNRNPK is HCV genotype independent. Forty-eight hours post silencing, siRNA-transfected cells were infected with JcR2a (A) or H77R2a (B) or Con1R2a (C) reporter virus (MOI = 0.4 TCID50/ml). To determine the impact of knock-down on viral entry/replication (grey bars), cells were lysed 48 h post infection. To measure knock-down impact on virus production (black bars), supernatants of transfected cells were used to inoculate Huh7.5 cells that were lysed 72 h later. Virus replication was quantified by measuring Renilla luciferase activity (relative light units, RLU) and values were normalized to cell viability. Non-targeting control siRNA (siContr.) was set to 100% (dotted line). SiRNAs targeting the Renilla luciferase sequence in the reporter virus genomes served as positive control. Bars represent the mean ±SD of at least two independent experiments. (D) HNRNPK knock-down also enhances production of JFH-1 wildtype virus. Huh7.5 cells were co-electroporated with JFH-1 RNA and either a mix of HNRNPK-specific siRNAs (#1–4) or a control siRNA. After 72 h titers of infectious virus contained in the supernatant (extracellular) or cell lysate (intracellular) were determined by TCID50 assay. Shown is the average of three independent experiments ±SD. Knock down efficiency was determined by Western blot; a representative blot is shown. In all panels, statistical analysis was performed by using Student's t-test, with reference to siContr. ***, P-value ≤0.0005; **, P-value ≤0.005; *, P-value ≤0.05; ns, non-significant. (TIF) [file ppat.1004573.s004.tif]

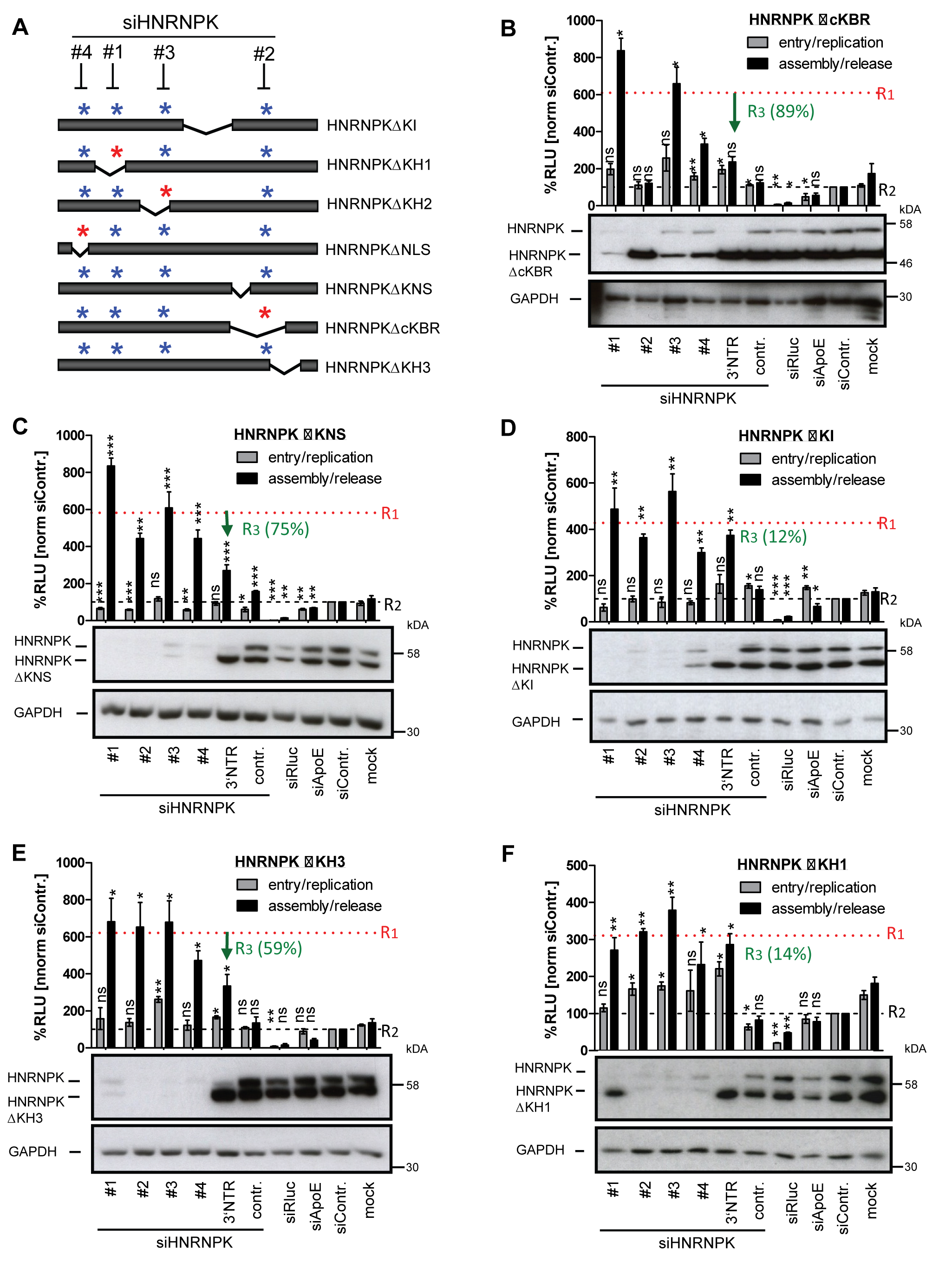

Supplement: S5 Fig — Mapping of HNRNPK domains required for restriction of HCV particle production. (A) Schematic of siRNA targeting sites in HNRNPK deletion mutants. Target sites of siHNRNPK#1 to #4 are indicated with an asterisk. Blue and red color indicate present or absent target sites, respectively. (B-F) Characterization of HNRNPK mutants ΔcKBR, ΔKNS, ΔKI, ΔKH3 or ΔKH1 for their efficiency to restrict HCV particle production, respectively. Silencing was performed by using 2.5 µM of each indicated siRNA. Cells were infected with the HCV Renilla luciferase reporter virus JcR2a (MOI = 0.4 TCID50/cell) after a 48 h-silencing period. To detect cellular genes involved in HCV entry/replication (grey bars), cells were lysed 48 h post infection and Renilla luciferase activity was measured. To evaluate the effect of silencing on assembly/release, virus-containing supernatant was used for infection of naïve Huh7.5 cells (black bars). Replication was determined by Renilla luciferase assay in lysates of cell prepared 72 h post inoculation. Values (relative light units, RLU) were normalized to cell viability and the non-targeting control siRNA (siContr.) that was set to 100% (R2 value, black dotted line). Bars represent the mean ±SD of four independent experiments. To quantify suppression of virus production by ectopic HNRNPK expression, mean values obtained with siRNA HNRNPK#1 to #4 (dotted horizontal red line; value R1) and siHNRNPK-3′NTR targeting only endogenous HNRNPK were compared with each other (value R3). Values were normalized to the ones obtained with the siContr. that was set 100% (value R2; dotted horizontal black line). Thus, R3 values reflect the relative suppression of HCV particle production achieved with ectopically expressed HNRNPK. ***, P-value ≤0.0005; **, P-value ≤0.005; *, P-value ≤0.05; ns, non-significant. Statistical analysis was performed by using Student's t-test, referred to the non-targeting control siRNA (siContr.). (TIF) [file ppat.1004573.s005.tif]

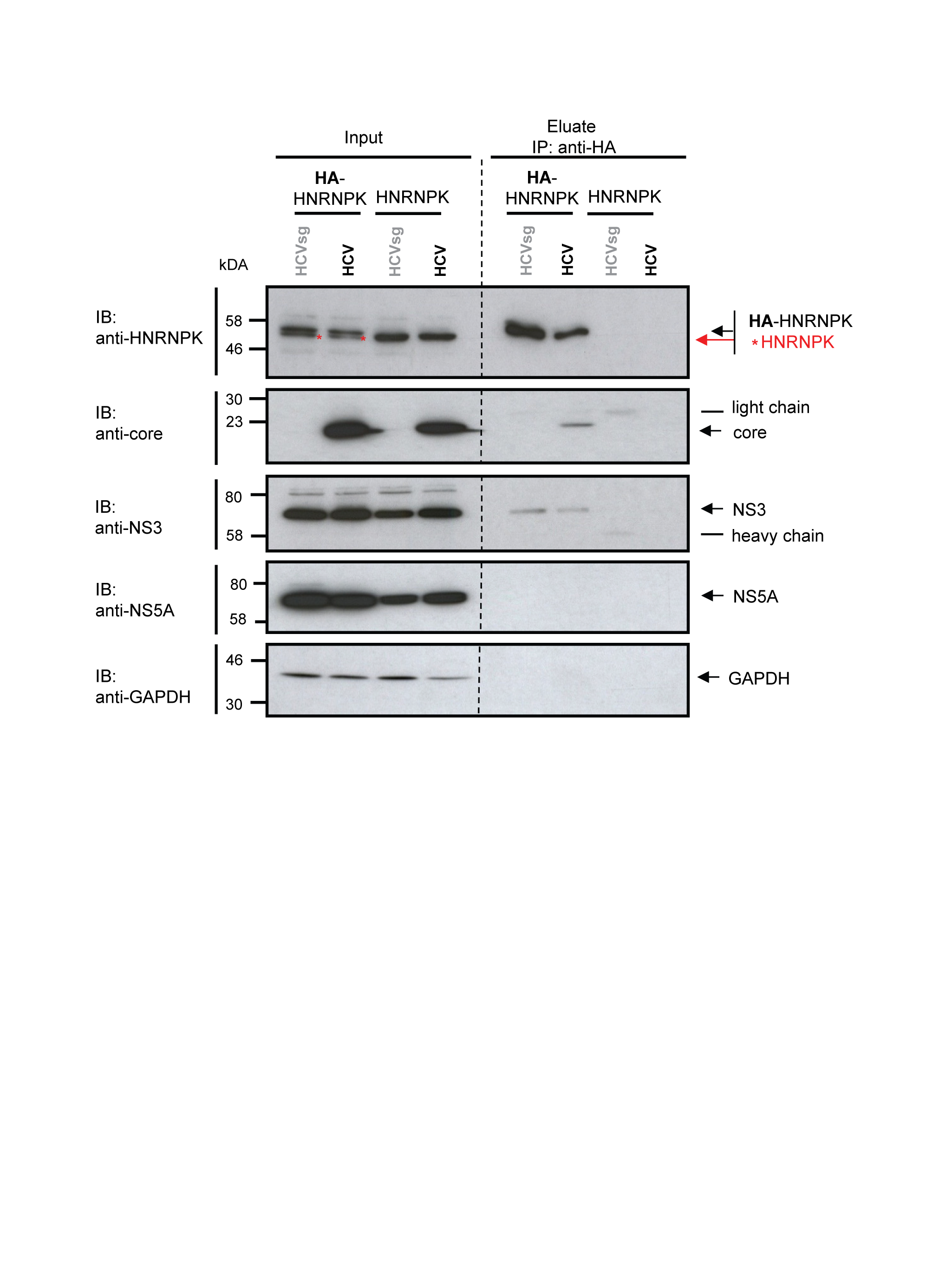

Supplement: S6 Fig — Interaction of HNRNPK with HCV core and NS3. Huh7.5 cells stably expressing wild type or HA-tagged HNRNPK were electroporated with a subgenomic JFH1 replicon (HCVsg) or genomic HCV RNA (HCV). Cells were harvested 48 h post electroporation and lysates were used for coimmunoprecipitation using HA-specific antibody-coated agarose beads. Immunocomplexes and total cell lysates (corresponding to 10% and 1% of the total fraction, respectively) were analyzed by immunoblot (IB) using antibodies specified in the left. GAPDH was used as loading control. Endogenous HNRNPK is indicated with red asterisks. Numbers in the left refer to the positions of molecular weight protein standards. (TIF) [file ppat.1004573.s006.tif]

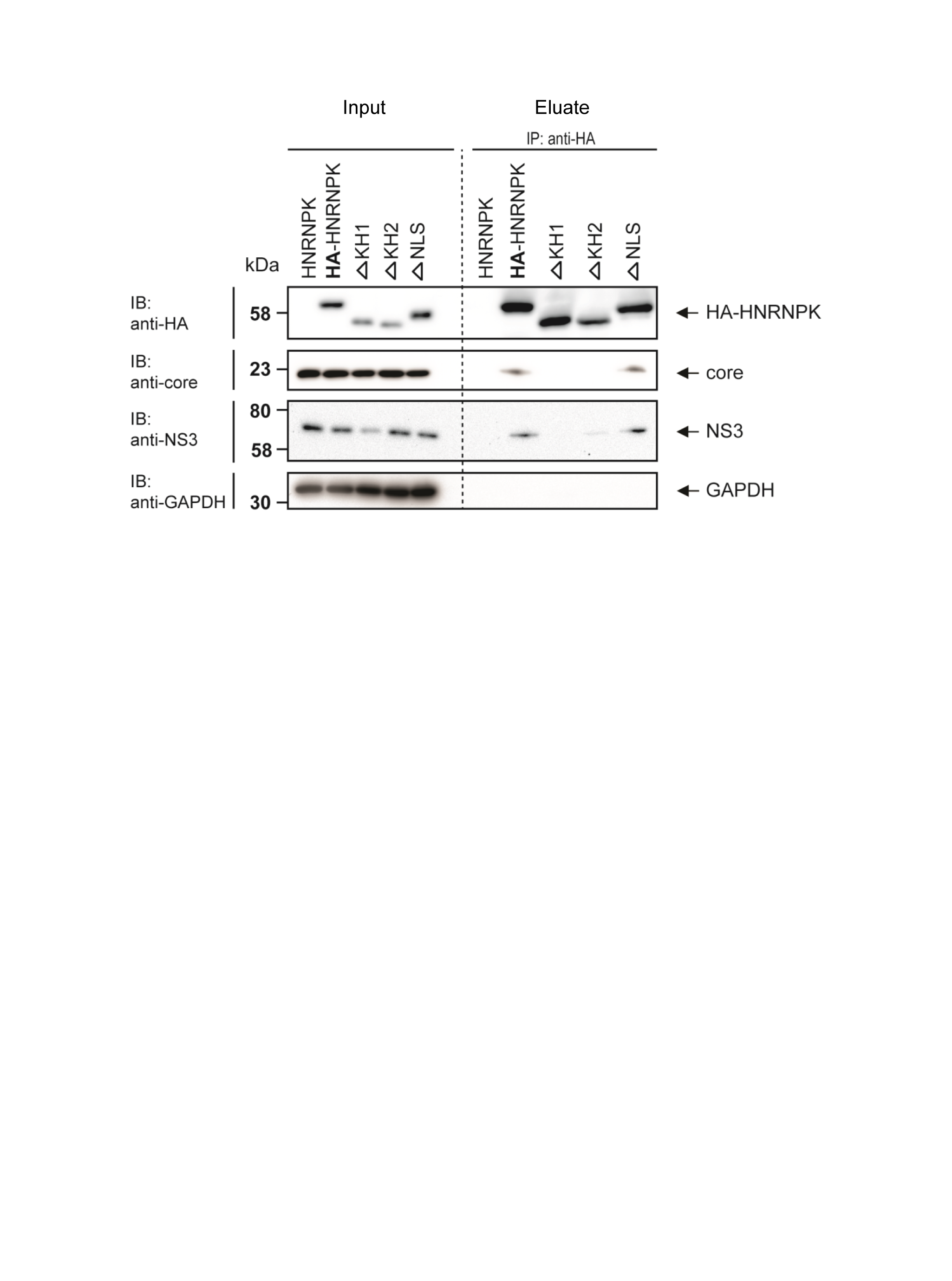

Supplement: S7 Fig — Interaction of HNRNPK deletion mutants with HCV core and NS3. Huh7.5 cells stably expressing wild type, HA-tagged HNRNPK or HA-tagged HNRNPK deletion mutants (HNRNPKΔKH1, ΔKH2 and ΔNLS) were electroporated with 5 µg genomic HCV RNA. Cells were harvested 48 h later and lysates were used for coimmunoprecipitation using HA-specific antibody-coated agarose beads. Immunocomplexes and total cell lysates (corresponding to 10% and 1% of the total fraction, respectively) were analyzed by immunoblot (IB) using antibodies specified in the left. GAPDH was used as loading control. Numbers in the left refer to the positions of molecular weight protein standards. (TIF) [file ppat.1004573.s007.tif]

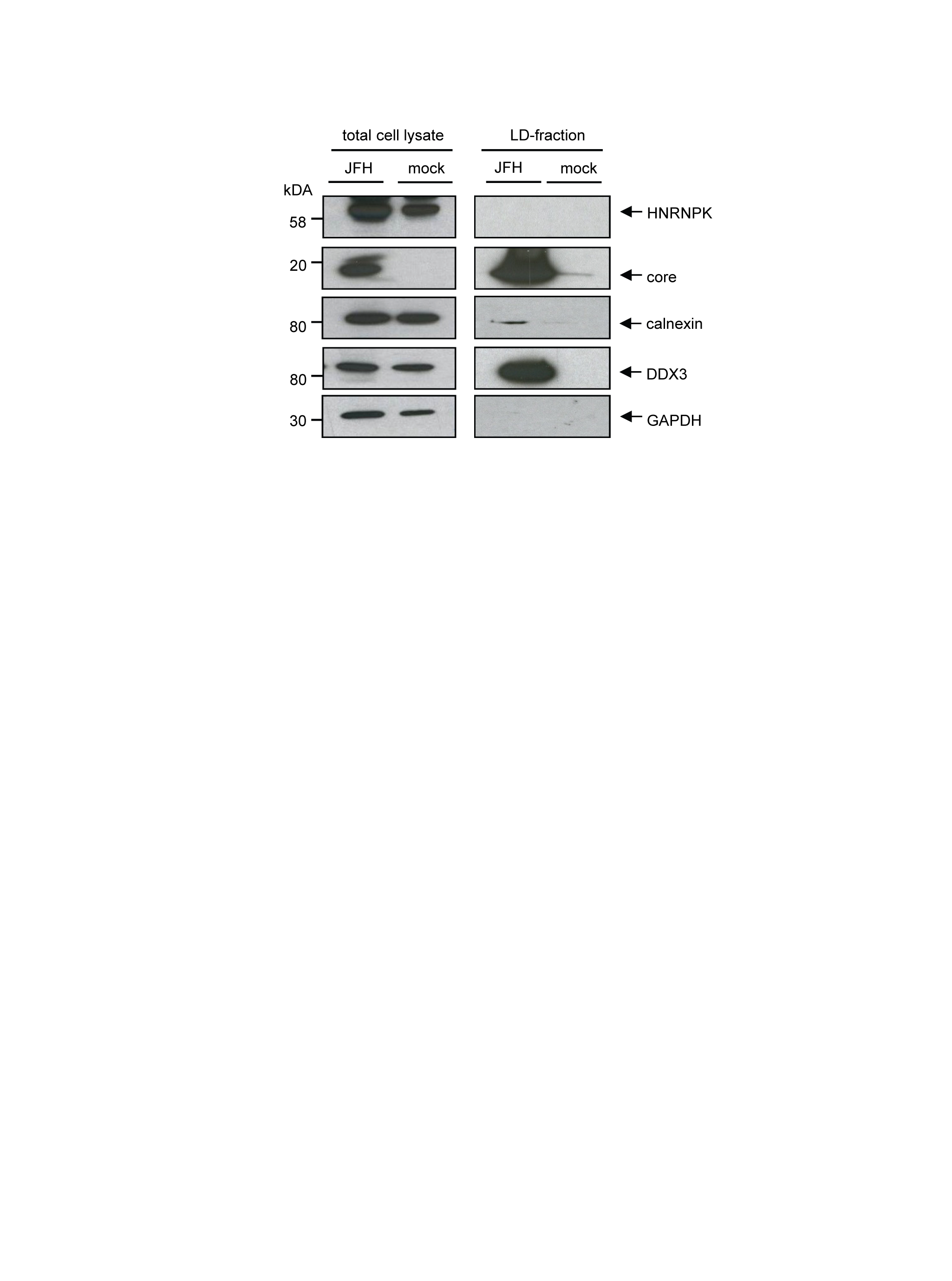

Supplement: S8 Fig — HNRNPK does not associate with LDs in HCV-containing cells. Huh7.5 cells were electroporated with genomic JFH1 (HCV) RNA or mock-transfected. Cells were harvested 72 h later and lipid droplets (LD) were isolated from total cell lysate by differential centrifugation. Total cell lysate as well as LD-fractions were analyzed by Western blot using antibodies specified in the right. Purity of LD fractions was determined by using antibodies detecting core, calnexin, DDX3 and GAPDH. (TIF) [file ppat.1004573.s008.tif]

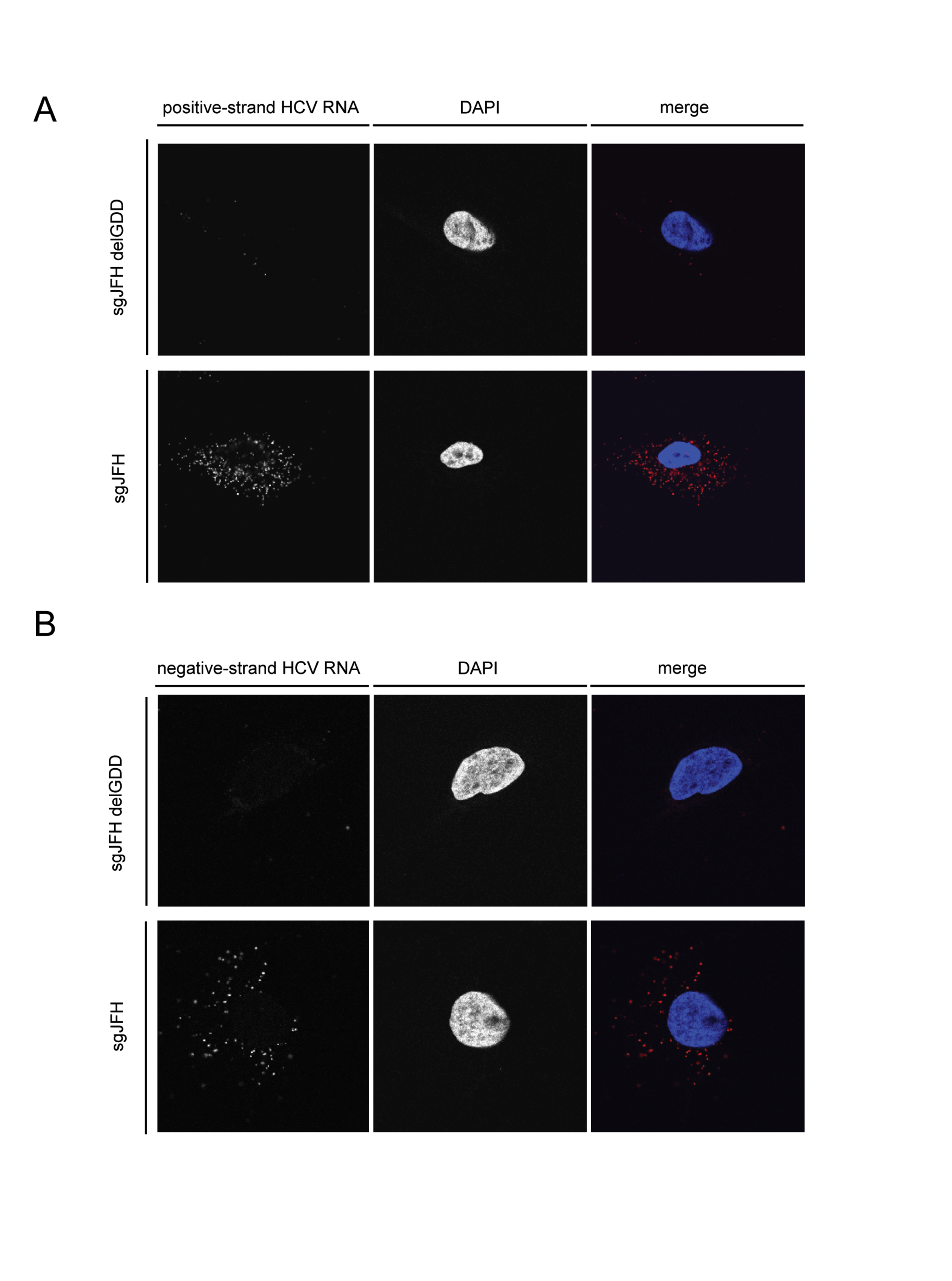

Supplement: S9 Fig — Specificity of HCV positive-stand and negative-strand RNA detection. (A, B) Huh7hp cells were electroporated with either 5 µg subgenomic JHF1 RNA or a replication-deficient JFH1 mutant containing mutations at the NS5B polymerase active site (sgJFHdelGDD). Seventy two hours after transfection, cells were fixed and HCV RNA was detected by fluorescent in situ hybridization (FISH) using the QuantiGene ViewRNA ISH Cell Assay (Affymetrix). Nuclear DNA was stained with DAPI. Note that Huh7hp cells were used instead of Huh7.5 cells because of the lower background staining obtained by FISH. (A) Positive-strand HCV RNA detection; B) Negative-strand HCV RNA detection. (TIF) [file ppat.1004573.s009.tif]

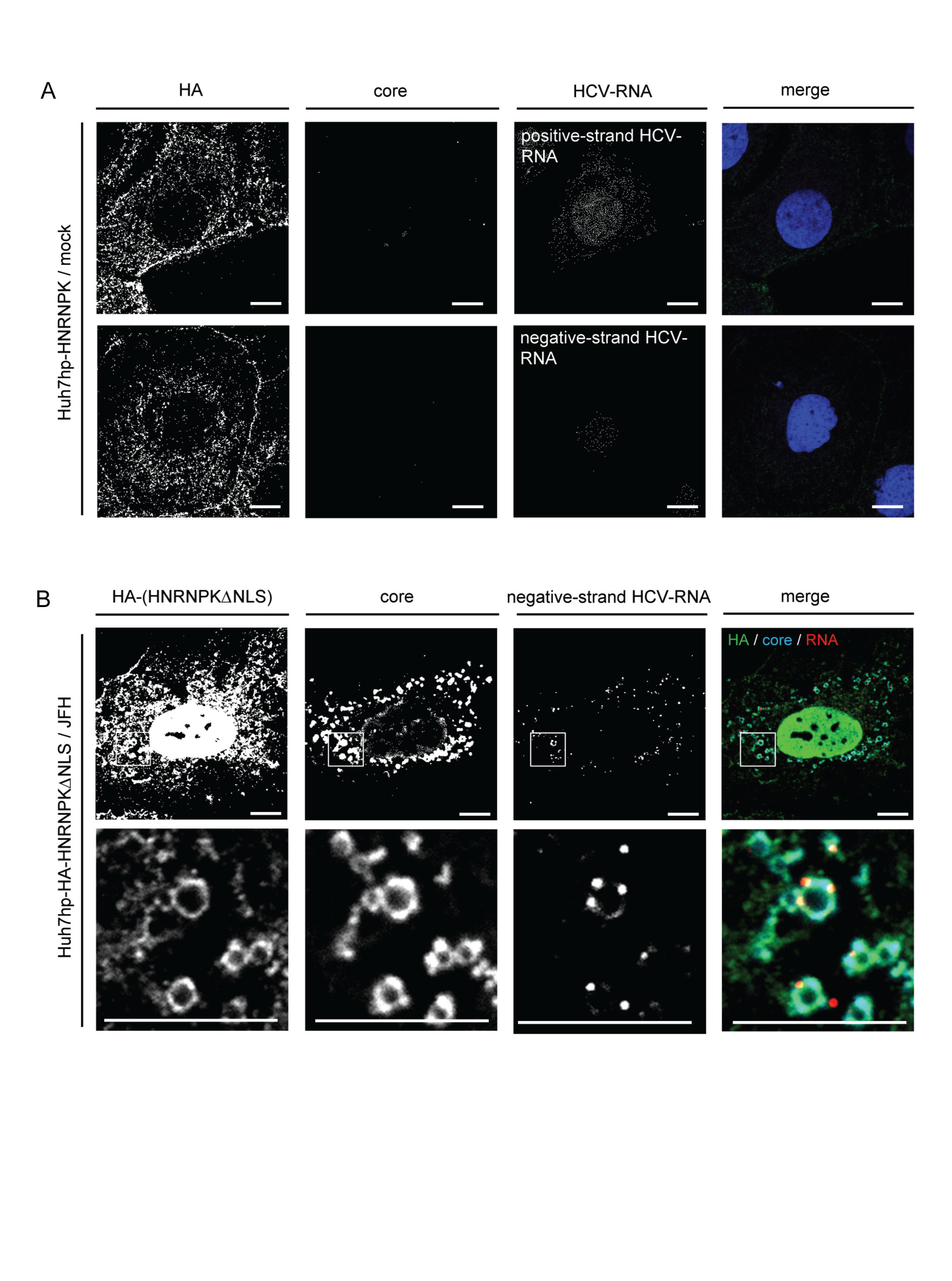

Supplement: S10 Fig — Quality control of strand-specific detection of HCV RNA and colocalization of HNRNPK with core and viral RNA. (A) Huh7hp cells stably expressing untagged HNRNPK were mock-electroporated and stained for the HA-tag, core as well as positive- and negative-strand HCV-RNA. Seventy two hours after transfection, cells were fixed and stained. Nuclear DNA was stained with DAPI. Note the absence of background staining. Note that Huh7hp cells were used instead of Huh7.5 cells because of the lower background staining obtained by FISH. (B) Huh7hp cells stably expressing HA-tagged HNRNPKΔNLS were electroporated with 5 µg genomic JFH1 (HCV) RNA and fixed 72 h later. Tagged HNRNPKΔNLS was detected by HA-specific immunofluorescence staining. HCV negative-strand RNA was detected by FISH using the QuantiGene ViewRNA ISH Cell Assay (Affymetrix). Enlargements of the sections shown in the bottom are indicated by white squares in the corresponding top panels. Images were acquired with a confocal microscope; scale bar refers to 10 µm. (TIF) [file ppat.1004573.s010.tif]

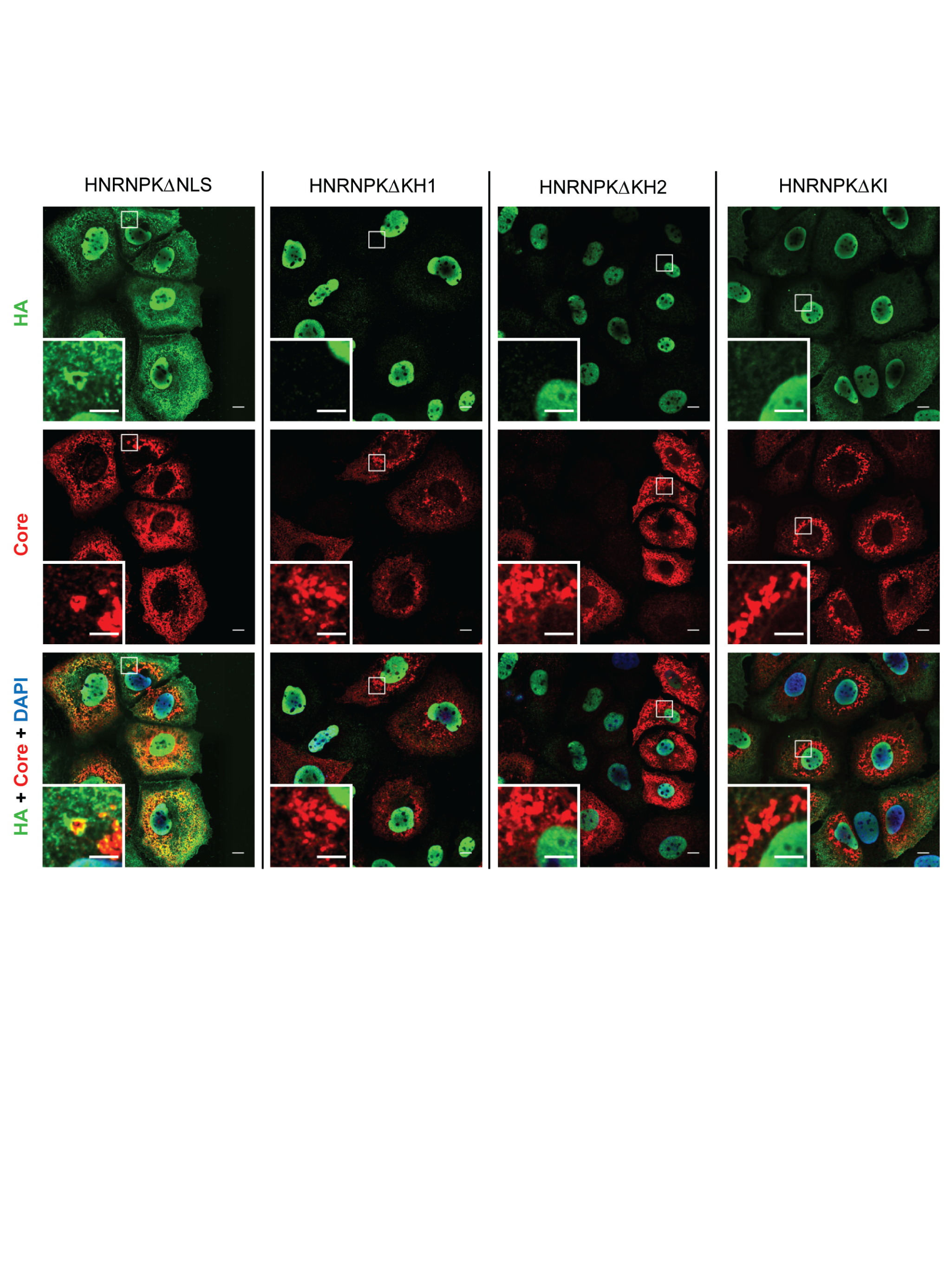

Supplement: S11 Fig — Subcellular distribution of HNRNPK deletion mutants in HCV-containing cells. Huh7hp cells stably expressing the HA-tagged variants HNRNPKΔNLS, HNRNPKΔKH1, HNRNPKΔKH2 or HNRNPKΔKI were electroporated with genomic JFH1 RNA and fixed 48 h later. HA-tagged HNRNPK variants were detected by immunofluorescence staining using a HA-specific antibody and Core was detected by using a monoclonal antibody. Inserts in the lower left of the images show magnifications of boxed areas. Scale bars represent 10 µm or 5 µm in regular and magnified images, respectively. (TIF) [file ppat.1004573.s011.tif]
