# Supplementary material for: Identification of HNRNPK as Regulator of Hepatitis C Virus Particle Production
Source: PLoS Pathog. 2015 Jan 8;11(1):e1004573. doi: 10.1371/journal.ppat.1004573 (PMC4287573; doi:10.1371/journal.ppat.1004573)
Supplement: S1 Methods — Supplemental Materials and Methods. (DOC) [file ppat.1004573.s014.doc]

**Supplemental information**

**Supplemental Materials and Methods**

**High-throughput siRNA screen.** The siRNA library used for the primary siRNA screen (Ambion Silencer Extended druggable genome library V3) contains a total of 27306 siRNAs to target a specific subset of 9,102 human genes (listed in Table S2A and B), with three independent siRNAs per gene. Production of 384- (primary screen) and 96-well plates (validation screen) for solid-phase reverse transfection has been described earlier [1]. In brief, to prepare 384-well plates 3 μl OptiMEM (supplemented with 0.4 M sucrose), 3.5 μl Lipofectamine 2000 (Invitrogen, Darmstadt, Germany) and 5 μl of the respective siRNA stock solution (30 μM) were mixed using an automated liquid handler (Microlab STAR, Hamilton, Martinsried, Germany) in 384-well low-volume plates (Nalge Nunc International) and incubated for 30 min at RT. Afterwards, 7.25 μl of a 0.2% (w/v) gelatin solution containing 0.01% (v/v) fibronectin was added and 18 μl of the mix was diluted in 180 μl ddH2O. Five μl of the transfection mix was pipetted into each well of a white μclear 384-well plate (Greiner Bio-One, Frickenhausen, Germany) and dried in a centrifugal evaporator (Genevac Mivac Quattro, Thermo-Fisher, Schwerte, Germany). For the validation siRNA screen, 96-well plates were prepared in the analogous way with minor modifications. Briefly. 2.38 μl OptiMEM (supplemented with 0.4 M sucrose), 0.87 μl Lipofectamine 2000 (Invitrogen, Darmstadt, Germany) and 2.5 μl of the respective siRNA stock solution (15 μM, ONTARGETplus, Dharmacon, Schwerte, Germany) were mixed using an automated liquid handler (Microlab STAR, Hamilton, Martinsried, Germany) in 384-well low-volume plates (Nalge Nunc International) and incubated for 30 min at RT. Afterwards, 3.625 μl of a 0.2% (w/v) gelatine solution containing 0.01% (v/v) fibronectin was added and 9 μl of the resulting mix was diluted in 450 μl ddH2O. Fifty μl of the transfection mix was pipetted into each well of a white μclear 96-well plate (Greiner Bio-One, Frickenhausen, Germany) and dried in a centrifugal evaporator (Genevac Mivac Quattro).

*Cell seeding, infection and reinfection.* For the primary screen 1.5 x 10^3^ Huh7.5 FLuc cells were seeded per siRNA-coated well of a 384-well plate in a volume of 30 µl. For the validation siRNA screen, 5 x 10^3^ Huh7.5 FLuc cells were seeded per well of a siRNA-coated 96-well plate in a volume of 200 µl. After 42 h cells were infected with the JcR2a using a MOI of ~1 TCID_50_/cell and medium was exchanged 24 h later. Forty-eight hours post infection cells were washed once with PBS, lysed in luciferase lysis buffer using 20 µl per well of a 384-well plate or 30 µl per well in case of a 96-well plate, respectively. To determine infectivity titers in supernatants of primary infected cells, naïve Huh7.5 FLuc cells were inoculated with culture supernatants, thus allowing identification of host cell factors involved in HCV assembly and release. Seventy-two hours later, viral replication was quantified by *Renilla* luciferase assay. Cytotoxicity was monitored by measuring *Firefly* luciferase activity. The primary screen was repeated three times; the validation screen four times.

**Statistical analysis of the screen.** Data were normalized in R/Bioconductor using the RNAither package [2]. After exclusion of lowest and highest 5% of wells based on cell counts, signal intensities were normalized for cell count effects using locally weighted scatterplot smoothing. We then performed within-plate and between-plate normalization using the Bscore method as implemented in RNAither for the primary screen, using the full screen as a quasi-negative control, and by zscore-normalization using the median and median absolute deviation of the negative controls as reference for the validation screens. Replicates were summarized using the mean. Hits were defined based on a score threshold of +/- 2 for at least two siRNAs per gene.

**Bioinformatic resources.** Cellular factors were extracted from the respective publications [3–21]. Human protein-protein interactions were taken from iRefIndex [22], which consolidates data from the Biomolecular Interaction Network Database (BIND) [23], BioGRID [24], CORUM [25], the Database of Interacting Proteins (DIP) [26], the Human Protein Reference Database (HPRD) [27], InnateDB [28], IntAct [29], MatrixDB [30], the MIPS mammalian protein-protein interaction database [31] and the Molecular INTeraction database (MINT) [32]. Protein complexes and predicted protein interactions were excluded. Interactions between human and HCV proteins were taken from [33]. Functional protein annotations of the categories biological process (BP), molecular function (MF) and cellular component (CC) were retrieved from the Gene Ontology (GO) [34] version 2013.06. UniProtKB keywords were obtained via DAVID v6.7 [35].

For all computational analyses, the various protein and gene identifiers were unified to NCBI gene identifiers. Entries without a valid gene mapping were excluded, entries with multiple mappings were merged. GO biological process enrichments were computed with TopGO [36], version 2.12 that is bundled to Bioconductor 2.12, using a LEA score < 0.001 as significance treshold. Network visualizations were created in Cytoscape [37].

**Bioinformatic analysis.** To reveal cellular processes critically involved in the HCV life cycle, we performed an integrative computational analysis. We first identified cellular interaction partners of our confirmed hits that were described in other HCV siRNA screens (Figure 2 and Table S1F). This extended pool of host factors was analyzed for significantly over-represented GO biological processes (Table S1D). In this way we identified intracellular protein transport pathways such as the COP-I system as one of the most prominent pathways involved in the HCV life cycle, which is consistent with an earlier siRNA-based study [4]. Additionally enriched factors included the epidermal growth factor receptor signaling pathway consistent with its role in HCV entry [38], signal recognition particle receptor-dependent transport and signal peptide processing (reviewed in [39]) or the LDL-pathway, consistent with the tight link of HCV assembly with intracellular lipid synthesis and storage systems [40].

**Meta-analysis of HCV relevant host cell factors.** The following data sources were implemented into the meta-analysis: i) HCV siRNA screens with genome-wide or selected siRNA libraries [3–8,10–21]; ii) genome-wide high-throughput yeast-two hybrid protein interaction study [33]; iii) comparative analysis of the proteome of crude replication complexes (CRCs) i.e. membrane preparations containing active HCV replicase complexes (host cell factor enrichment in CRC preparations relative to identical membrane preparations from naïve cells, and the resistance of CRC-associated host cell factors to proteinase K digestion [41–43], data sets generated in our laboratory); iv) comparative transcriptome analyses between naïve Huh7 cells and low / highly permissive Huh7 cells [44,45] (assuming that the latter express higher amounts of HCV-specific HDFs) and of mouse hepatocytic cells (Hep56.1D) or human HuH6 cells with or without a subgenomic HCV replicon [46,47] (assuming that host factors of relevance for HCV might be expressed differentially in the presence of replicons); v) our own primary HCV siRNA primary screen.

**References to supplemental information**

1. Erfle H, Neumann B, Rogers P, Bulkescher J, Ellenberg J, et al. (2008) Work flow for multiplexing siRNA assays by solid-phase reverse transfection in multiwell plates. J Biomol Screen 13: 575-580.

2. Rieber N, Knapp B, Eils R, Kaderali L (2009) RNAither, an automated pipeline for the statistical analysis of high-throughput RNAi screens. Bioinformatics 25: 678-679.

3. Li Q, Brass AL, Ng A, Hu Z, Xavier RJ, et al. (2009) A genome-wide genetic screen for host factors required for hepatitis C virus propagation. Proc Natl Acad Sci U S A 106: 16410-16415.

4. Tai AW, Benita Y, Peng LF, Kim SS, Sakamoto N, et al. (2009) A functional genomic screen identifies cellular cofactors of hepatitis C virus replication. Cell Host Microbe 5: 298-307.

5. Randall G, Panis M, Cooper JD, Tellinghuisen TL, Sukhodolets KE, et al. (2007) Cellular cofactors affecting hepatitis C virus infection and replication. Proc Natl Acad Sci U S A 104: 12884-12889.

6. Berger KL, Cooper JD, Heaton NS, Yoon R, Oakland TE, et al. (2009) Roles for endocytic trafficking and phosphatidylinositol 4-kinase III alpha in hepatitis C virus replication. Proc Natl Acad Sci U S A106: 7577-75782. .

7. Reiss S, Rebhan I, Backes P, Romero-Brey I, Erfle H, et al. (2011) Recruitment and activation of a lipid kinase by hepatitis C virus NS5A is essential for integrity of the membranous replication compartment. Cell Host Microbe 9: 32-45.

8. Supekova L, Supek F, Lee J, Chen S, Gray N, et al. (2008) Identification of human kinases involved in hepatitis C virus replication by small interference RNA library screening. J Biol Chem 283: 29-36.

9. Ng TI, Mo H, Pilot-Matias T, He Y, Koev G, et al. (2007) Identification of host genes involved in hepatitis C virus replication by small interfering RNA technology. Hepatology 45: 1413-1421.

10. Vaillancourt FH, Pilote L, Cartier M, Lippens J, Liuzzi M, et al. (2009) Identification of a lipid kinase as a host factor involved in hepatitis C virus RNA replication. Virology 387: 5-10.

11. Borawski J, Troke P, Puyang X, Gibaja V, Zhao S, et al. (2009) Class III phosphatidylinositol 4-kinase alpha and beta are novel host factor regulators of hepatitis C virus replication. J Virol 83: 10058-10074.

12. Trotard M, Lepere-Douard C, Regeard M, Piquet-Pellorce C, Lavillette D, et al. (2009) Kinases required in hepatitis C virus entry and replication highlighted by small interference RNA screening. FASEB J 23: 3780-3789.

13. Jones DM, Domingues P, Targett-Adams P, McLauchlan J (2010) Comparison of U2OS and Huh-7 cells for identifying host factors that affect hepatitis C virus RNA replication. J Gen Virol 91: 2238-2248.

14. Coller KE, Berger KL, Heaton NS, Cooper JD, Yoon R, et al. (2009) RNA interference and single particle tracking analysis of hepatitis C virus endocytosis. PLoS Pathog 5: e1000702.

15. Coller KE, Heaton NS, Berger KL, Cooper JD, Saunders JL, et al. (2012) Molecular determinants and dynamics of hepatitis C virus secretion. PLoS Pathog 8: e1002466.

16. Lupberger J, Zeisel MB, Xiao F, Thumann C, Fofana I, et al. (2011) EGFR and EphA2 are host factors for hepatitis C virus entry and possible targets for antiviral therapy. Nat Med 17: 589-595.

17. Chen YC, Su WC, Huang JY, Chao TC, Jeng KS, et al. (2010) Polo-like kinase 1 is involved in hepatitis C virus replication by hyperphosphorylating NS5A. J Virol 84: 7983-7993.

18. Chao TC, Su WC, Huang JY, Chen YC, Jeng KS, et al. (2012) Proline-serine-threonine phosphatase-interacting protein 2 (PSTPIP2), a host membrane-deforming protein, is critical for membranous web formation in hepatitis C virus replication. J Virol 86: 1739-1749.

19. Hara H, Aizaki H, Matsuda M, Shinkai-Ouchi F, Inoue Y, et al. (2009) Involvement of creatine kinase B in hepatitis C virus genome replication through interaction with the viral NS4A protein. J Virol 83: 5137-5147.

20. Herker E, Harris C, Hernandez C, Carpentier A, Kaehlcke K, et al. (2010) Efficient hepatitis C virus particle formation requires diacylglycerol acyltransferase-1. Nat Med 16: 1295-1298.

21. Xue Q, Ding H, Liu M, Zhao P, Gao J, et al. (2007) Inhibition of hepatitis C virus replication and expression by small interfering RNA targeting host cellular genes. Arch Virol 152: 955-962.

22. Razick S, Magklaras G, Donaldson IM (2008) iRefIndex: a consolidated protein interaction database with provenance. BMC Bioinformatics 9: 405. 1471-2105.

23. Alfarano C, Andrade CE, Anthony K, Bahroos N, Bajec M, et al. (2005) The Biomolecular Interaction Network Database and related tools 2005 update. Nucleic Acids Res 33: D418-D424.

24. Chatr-Aryamontri A, Breitkreutz BJ, Heinicke S, Boucher L, Winter A, et al. (2013) The BioGRID interaction database: 2013 update. Nucleic Acids Res 41: D816-D823.

25. Ruepp A, Waegele B, Lechner M, Brauner B, Dunger-Kaltenbach I, et al. (2010) CORUM: the comprehensive resource of mammalian protein complexes--2009. Nucleic Acids Res 38: D497-D501.

26. Salwinski L, Miller CS, Smith AJ, Pettit FK, Bowie JU, et al. (2004) The Database of Interacting Proteins: 2004 update. Nucleic Acids Res 32: D449-D451.

27. Keshava Prasad TS, Goel R, Kandasamy K, Keerthikumar S, Kumar S, et al. (2009) Human Protein Reference Database--2009 update. Nucleic Acids Res 37: D767-D772.

28. Lynn DJ, Winsor GL, Chan C, Richard N, Laird MR, et al. (2008) InnateDB: facilitating systems-level analyses of the mammalian innate immune response. Mol Syst Biol 4: 218.

29. Kerrien S, Aranda B, Breuza L, Bridge A, Broackes-Carter F, et al. (2012) The IntAct molecular interaction database in 2012. Nucleic Acids Res 40: D841-D846.

30. Chautard E, Fatoux-Ardore M, Ballut L, Thierry-Mieg N, Ricard-Blum S (2011) MatrixDB, the extracellular matrix interaction database. Nucleic Acids Res 39: D235-D240.

31. Pagel P, Kovac S, Oesterheld M, Brauner B, Dunger-Kaltenbach I, et al. (2005) The MIPS mammalian protein-protein interaction database. Bioinformatics 21: 832-834.

32. Licata L, Briganti L, Peluso D, Perfetto L, Iannuccelli M, et al. (2012) MINT, the molecular interaction database: 2012 update. Nucleic Acids Res 40: D857-D861.

33. de Chassey B, Navratil V, Tafforeau L, Hiet MS, Aublin-Gex A, et al. (2008) Hepatitis C virus infection protein network. Mol Syst Biol 4: 230.

34. The Gene Ontology in 2010: extensions and refinements. (2012) Nucleic Acids Res 38:

35. Huang dW, Sherman BT, Lempicki RA (2009) Systematic and integrative analysis of large gene lists using DAVID bioinformatics resources. Nat Protoc 4: 44-57.

36. Alexa A, Rahnenfuhrer J, Lengauer T (2006) Improved scoring of functional groups from gene expression data by decorrelating GO graph structure. Bioinformatics 22: 1600-1607.

37. Smoot ME, Ono K, Ruscheinski J, Wang PL, Ideker T (2011) Cytoscape 2.8: new features for data integration and network visualization. Bioinformatics 27: 431-432.

38. Zona L, Lupberger J, Sidahmed-Adrar N, Thumann C, Harris HJ, et al. (2013) HRas signal transduction promotes hepatitis C virus cell entry by triggering assembly of the host tetraspanin receptor complex. Cell Host Microbe 13: 302-313.

39. Moradpour D, Penin F (2013) Hepatitis C virus proteins: from structure to function. Curr Top Microbiol Immunol 369: 113-142.

40. Andre P, Komurian-Pradel F, Deforges S, Perret M, Berland JL, et al. (2002) Characterization of low- and very-low-density hepatitis C virus RNA-containing particles. J Virol 76: 6919-6928.

41. MacPherson JI, Sidders B, Wieland S, Zhong J, Targett-Adams P, et al. (2011) An integrated transcriptomic and meta-analysis of hepatoma cells reveals factors that influence susceptibility to HCV infection. PLoS ONE 6: e25584.

42. Miyanari Y, Hijikata M, Yamaji M, Hosaka M, Takahashi H, et al. (2003) Hepatitis C virus non-structural proteins in the probable membranous compartment function in viral genome replication. J Biol Chem 278: 50301-50308.

43. Quinkert D, Bartenschlager R, Lohmann V (2005) Quantitative analysis of the hepatitis C virus replication complex. J Virol 79: 13594-13605.

44. Binder M, Kochs G, Bartenschlager R, Lohmann V (2007) Hepatitis C virus escape from the interferon regulatory factor 3 pathway by a passive and active evasion strategy. Hepatology 46: 1365-1374.

45. Binder M, Sulaimanov N, Clausznitzer D, Schulze M, Huber CM, et al. (2013) Replication vesicles are load- and choke-points in the hepatitis C virus lifecycle. PLoS Pathog 9: e1003561.

46. Windisch MP, Frese M, Kaul A, Trippler M, Lohmann V, et al. (2005) Dissecting the interferon-induced inhibition of hepatitis C virus replication by using a novel host cell line. J Virol 79: 13778-13793.

47. Long G, Hiet MS, Windisch MP, Lee JY, Lohmann V, et al. (2011) Mouse hepatic cells support assembly of infectious hepatitis C virus particles. Gastroenterology 141: 1057-1066.
